# The Performance of Wearable AI in Detecting Stress Among Students: Systematic Review and Meta-Analysis

**DOI:** 10.2196/52622

**Published:** 2024-01-31

**Authors:** Alaa Abd-alrazaq, Mohannad Alajlani, Reham Ahmad, Rawan AlSaad, Sarah Aziz, Arfan Ahmed, Mohammed Alsahli, Rafat Damseh, Javaid Sheikh

**Affiliations:** 1 AI Center for Precision Health Weill Cornell Medicine-Qatar Qatar Foundation Doha Qatar; 2 Institute of Digital Healthcare, WMG, University of Warwick Warwick United Kingdom; 3 Health Informatics Department, College of Health Science, Saudi Electronic University Riyadh Saudi Arabia; 4 Department of Computer Science and Software Engineering, United Arab Emirates University Al Ain, Abu Dhabi United Arab Emirates

**Keywords:** stress, artificial intelligence, wearable devices, machine learning, systematic review, students, mobile phone

## Abstract

**Background:**

Students usually encounter stress throughout their academic path. Ongoing stressors may lead to chronic stress, adversely affecting their physical and mental well-being. Thus, early detection and monitoring of stress among students are crucial. Wearable artificial intelligence (AI) has emerged as a valuable tool for this purpose. It offers an objective, noninvasive, nonobtrusive, automated approach to continuously monitor biomarkers in real time, thereby addressing the limitations of traditional approaches such as self-reported questionnaires.

**Objective:**

This systematic review and meta-analysis aim to assess the performance of wearable AI in detecting and predicting stress among students.

**Methods:**

Search sources in this review included 7 electronic databases (MEDLINE, Embase, PsycINFO, ACM Digital Library, Scopus, IEEE Xplore, and Google Scholar). We also checked the reference lists of the included studies and checked studies that cited the included studies. The search was conducted on June 12, 2023. This review included research articles centered on the creation or application of AI algorithms for the detection or prediction of stress among students using data from wearable devices. In total, 2 independent reviewers performed study selection, data extraction, and risk-of-bias assessment. The Quality Assessment of Diagnostic Accuracy Studies–Revised tool was adapted and used to examine the risk of bias in the included studies. Evidence synthesis was conducted using narrative and statistical techniques.

**Results:**

This review included 5.8% (19/327) of the studies retrieved from the search sources. A meta-analysis of 37 accuracy estimates derived from 32% (6/19) of the studies revealed a pooled mean accuracy of 0.856 (95% CI 0.70-0.93). Subgroup analyses demonstrated that the accuracy of wearable AI was moderated by the number of stress classes (*P*=.02), type of wearable device (*P*=.049), location of the wearable device (*P*=.02), data set size (*P*=.009), and ground truth (*P*=.001). The average estimates of sensitivity, specificity, and *F*_1_-score were 0.755 (SD 0.181), 0.744 (SD 0.147), and 0.759 (SD 0.139), respectively.

**Conclusions:**

Wearable AI shows promise in detecting student stress but currently has suboptimal performance. The results of the subgroup analyses should be carefully interpreted given that many of these findings may be due to other confounding factors rather than the underlying grouping characteristics. Thus, wearable AI should be used alongside other assessments (eg, clinical questionnaires) until further evidence is available. Future research should explore the ability of wearable AI to differentiate types of stress, distinguish stress from other mental health issues, predict future occurrences of stress, consider factors such as the placement of the wearable device and the methods used to assess the ground truth, and report detailed results to facilitate the conduct of meta-analyses.

**Trial Registration:**

PROSPERO CRD42023435051; http://tinyurl.com/3fzb5rnp

## Introduction

### Background

Students’ mental health is now of significant concern in the domain of public health. The academic environment, composed of rigorous curriculum structures, exams, assignments, evaluative metrics, deadlines, and an undercurrent of peer comparisons, not only requires a high cognitive effort but can also be a basis for psychosocial risks [1-3]. Being immersed in such scholarly endeavors at different levels, students become inadvertently vulnerable to mental health issues (eg, anxiety, depression, and stress [3,4]). This can negatively affect a student’s life in general and, specifically, their academic performance. Research has revealed that students who are constantly under high levels of stress are more inclined to underperform academically when compared with their peers who are less stressed [5]. Recent studies suggest that failure to manage academic-related stress is detrimental to the well-being of students [6-8].

The psychobiological literature defines stress, including academic stress, as a syndrome of responses combining interactions between the neurocognitive, endocrine, and affective systems [9-11]. Stress manifests when exposed to conditions that present a physical and psychological challenge or threat [11]. Stress is also defined as the result of incompatibility in relation to an individual’s ability to handle the burden placed on them by their environment [12]. Stress can be classified based on its duration into 2 forms: chronic and acute stress. Acute stress is a short-term stress response to an immediate situation and usually subsides soon after symptoms first appear, whereas chronic stress is a long-term stress response to ongoing stressors [13]. Typically, acute stress is the most common and does not impose a health burden on young and healthy individuals. In contrast, chronic stress can have a negative impact on both physical and mental health [14,15]. Potential biological risks include inflammation, diabetes, and heart disease [16,17]. Furthermore, studies have shown an association with cognitive dysfunctions because of excessive and chronic stressors [18,19]. Chronic stress can also have negative effects on social behavior, which leads to antisociality [20]; depression; or, in extreme cases, suicide [21,22]. Worldwide, it has been reported that, for every 5 visits by college students to their physician, 3 are for stress-related problems [23]. In addition, most college students who have problems with stress also have sleep-related disorders (76%), are prone to headaches (58%), experience difficult relationships with family and friends (85%), and tend to be short-tempered (70%) [23]. The American Institute of Stress reveals that, in the United States, a combined US $300 billion is spent on treating stress-related ailments and diseases [13].

Given the aforementioned complications of stress, the detection and monitoring of stress in early stages are crucial. Subjective and objective assessments can be used to evaluate stress levels. Subjective assessments of stress can involve 2 different means: standard self-reported questionnaires designed by field experts (eg, the Perceived Stress Scale and Depression, Anxiety, and Stress Scale–21 Items) or clinical interviews with psychologists [15,24]. Beyond this, objective assessments of stress include physical observations and physiological measures. For physical observations, visible changes in the appearance of the human body are noted, such as any facial expressions, the rate of blinking, and any noticeable dilation of the pupils. For physiological measures, blood, urine, or saliva samples can be collected to measure the level of specific hormones (adrenaline and cortisol) that are released as a response to a stressor [10,14,16]. Relying only on subjective assessments is insufficient to accurately detect and monitor stress as they are highly subjective, usually irreproducible, time-consuming, and entangled with other body responses not related to stress [25,26]. Although the aforementioned objective assessments can be deterministic in capturing stress status, they require invasive or obtrusive tests that are commonly deployed in a highly controlled laboratory environment and do not reflect stress in real-life scenarios [13,27]. Therefore, there is a critical need for alternative assessments of stress that are objective, noninvasive, nonobtrusive, and automatic and can continuously monitor biomarkers in real time.

Changes in biomarkers (eg, heart rate [HR], HR variability [HRV], and electrodermal activity [EDA]) can be used to objectively detect stress [15,28]. Real-time and continuous monitoring of these biomarkers must be supported by computational frameworks to automate the process of stress detection, thereby overcoming the aforementioned limitations of the current tools. Wearable artificial intelligence (AI) is a promising solution that has been used to address the issue of assessing stress [29-31]. Wearable AI is an advanced technology that depends on AI techniques to analyze a large amount of data (eg, HR, HRV, EDA, activity level, and skin temperature) collected by sensors in wearable devices to provide personalized feedback. Several types of wearable devices can be used to collect biomarkers: on-body devices, which are fixed directly on the body or skin; near-body devices, which are fixed close to the body but do not have direct contact with the skin; in-body devices, which are implanted in the body; and electronic textiles, which are fabrics that have integrated electronics [30].

### Research Problem and Aim

In the past few years, numerous studies have shed light on the performance of wearable AI for the detection of stress. Several reviews of such studies have been conducted, but they have the following limitations. First, none of the previous reviews have focused on students [13,15,24,28,32-35]. Second, none of the previous reviews have used statistical techniques (eg, meta-analysis) to analyze the results of previous studies [13,15,24,28,32-35]. Third, most of the previous reviews have been literature reviews rather than systematic reviews [13,15,24,28,33,34]. Fourth, the search sources in the previous reviews have not included the main databases in the field, such as MEDLINE [28], ACM Digital Library [28,32,33,35], Scopus [28,32,33,35], Embase [32,35], and IEEE Xplore [32,33,35]. Finally, several previous reviews have focused on wearable and nonwearable devices instead of on wearable devices only [13,15,24,32]. Therefore, this review sought to bridge this gap by assessing the capability of wearable AI to detect and forecast stress among students. Our research question is as follows: what is the effectiveness of wearable AI in diagnosing and predicting stress among students?

## Methods

### Overview

The authors undertook and reported this review in line with the guidelines set forth by the PRISMA-DTA (Preferred Reporting Items for Systematic Reviews and Meta-Analyses extension for Diagnostic Test Accuracy) [36]. The PRISMA-DTA checklist for this review is presented in Multimedia Appendix 1 [36]. The protocol for this review was registered with PROSPERO (ID: CRD42023435051).

### Search Strategy

To identify suitable studies, the primary author conducted a comprehensive search across 7 electronic repositories on June 12, 2023: MEDLINE (via Ovid), Embase (via Ovid), PsycINFO (via Ovid), ACM Digital Library, Scopus, IEEE Xplore, and Google Scholar. Using an automated search methodology, biweekly notifications were configured over a span of 3 months, concluding on September 12, 2023. Given the large number of results returned by Google Scholar, the first 100 results (equivalent to 10 pages) were subjected to scrutiny for this review. To find more studies, we checked the lists of sources referenced in the studies that we had already included (backward reference list checking), and we also looked at studies that had cited the ones we had already included (forward reference list checking).

The compilation of search terms for the review was informed by consultations with 2 digital mental health experts and scrutiny of relevant literature reviews. The resultant search query was composed of four distinct categories of terms: (1) terms associated with AI, such as “artificial intelligence,” “machine learning,” and “deep learning”; (2) terms linked to wearable devices, comprising “wearable*,” “smartwatch*,” and “smartband*”; (3) terms related to stress; and (4) terms relevant to students, including “student*,” “postgraduate*,” and “undergraduate*.” The Boolean OR operator was used to combine terms in the same category, whereas the Boolean AND operator was used to combine terms between categories. The precise search formulations are presented in Multimedia Appendix 2 for reference.

### Study Eligibility Criteria

This review analyzed research articles centered on the creation or application of AI algorithms for the detection or prediction of stress among students using data from wearable devices. The standards for determining which articles to include and exclude were jointly established through the collaborative expertise of the authors. To be eligible for consideration in this review, studies needed to collect data from students regardless of their educational level, age, gender, and ethnicity. Furthermore, to be included in this review, studies had to assess the effectiveness of AI algorithms in identifying or foreseeing stress. They were also required to present the confusion matrix or performance metrics (such as accuracy, sensitivity, and specificity). Studies that used AI to predict the outcomes of stress interventions or treatments were excluded.

Eligible studies were required to use noninvasive wearable devices (eg, smartwatches, smart glasses, and smart rings) for data collection. This review encompassed studies that used various methods for data collection (such as nonwearable devices, interviews, and questionnaires) in addition to wearable devices. In contrast, studies that solely used the following devices for data collection were excluded: nonwearable devices, handheld devices (eg, mobile phones), near-body wearable devices, in-body wearable devices, wearable devices wired to nonwearable devices, and wearables requiring expert oversight (such as those requiring precise electrode placement).

The inclusion criteria covered peer-reviewed journal articles, conference papers, preprints, and dissertations irrespective of the study setting, reference standard (ie, ground truth), or the country of study. Given the emphasis on contemporary technology and the continuous growth in the field of wearable AI, only articles from 2015 onward were included. Articles not in English or structured as review pieces, editorials, conference abstracts, posters, protocols, and research highlights were all excluded. We included only prospective and retrospective experimental studies; however, studies that used publicly available data sets (eg, Wearable Stress and Affect Detection [WESAD]) or data sets from previous studies were excluded.

### Study Selection

The study selection process involved 3 main steps. First, duplicate studies were removed using EndNote X9 (Clarivate Analytics). Then, 2 reviewers independently evaluated the titles and abstracts of the remaining articles. Finally, the reviewers independently assessed the full texts of the remaining articles. Any disagreements were discussed and resolved. The agreement between the reviewers was high, with a κ score of 0.87 for evaluating titles and abstracts and 0.92 for reading full texts.

### Data Extraction

The data extraction form was developed and tested with 3 studies first (Multimedia Appendix 3). Using Microsoft Excel (Microsoft Corp), 2 reviewers independently extracted the studies’ metadata, features of the wearable devices, features of the AI algorithms, and the study results. Any disagreements between the reviewers were resolved through discussion. For studies that provided raw data or confusion matrices, we calculated performance measures such as accuracy, specificity, and sensitivity. If this information was not available, we reached out to the first and corresponding authors of those studies to try to obtain it. We did not include results based only on nonwearable device data (eg, data from smartphones or surveys). As many studies conducted multiple experiments with different features, data types, validation methods, and AI algorithms, they reported several results for the same performance measure. In these cases, we obtained the best result in each performance measure for each algorithm.

### Risk-of-Bias and Applicability Appraisal

To assess the quality of the included studies, we tailored the established Quality Assessment of Diagnostic Accuracy Studies–Revised (QUADAS-2) [37] tool to suit the objectives of our review. We made adjustments by replacing certain nonapplicable criteria with more relevant ones from the Prediction Model Risk of Bias Assessment Tool [38]. Our adjusted QUADAS-2 tool includes 4 primary categories: “Participants,” “Index Test” (pertaining to AI algorithms), “Reference Standard” (denoting the ground truth), and “Analysis.” We formulated 4 specific questions within each category to align with the aims of our review. In addition to examining the potential biases within these 4 categories, we assessed the real-world applicability of the findings for the first 3 categories. To optimize our adjusted tool, we first trialed it on 3 studies to fine-tune it. In total, 2 reviewers independently assessed the included studies using the adapted QUADAS-2 tool (Multimedia Appendix 4). Discrepancies between their evaluations were discussed and resolved through consensus.

### Data Synthesis

The data from the studies were combined using narrative and statistical methods. In the narrative synthesis, we used text and tables to provide an overview and describe the key features of the included studies (study metadata, wearable devices, and AI techniques). A statistical approach was used when at least 2 different studies presented sufficient data to conduct meta-analyses. We did not consider the study design when selecting studies for meta-analysis. When estimates (eg, accuracy, sensitivity, and specificity) in the analysis were extracted from different unique studies (ie, independent effect sizes), DerSimonian-Laird random-effects models [39] using the Freeman-Tukey double arcsine transformation [40,41] were conducted to pool the extracted estimates. This approach considers the fluctuations arising from sampling and the heterogeneity in estimates. The analysis was executed using the *meta* toolkit in R (version 4.2.2; R Foundation for Statistical Computing) [42]. However, when there were estimates in the analysis extracted from the same study (ie, dependent effect sizes), we used a multilevel meta-analysis technique [39,43] to account for this dependency in effect sizes, thereby reducing the likelihood of type-I errors. Multilevel meta-analyses were conducted using the *metafor* toolkit in R (version 4.2.2) [40].

Where appropriate, we performed subgroup multilevel meta-analyses to investigate potential relationships between performance estimates and various factors [39,43]. These factors included AI algorithms, number of stress classes, type of wearable device, location of the wearable device, data set size, data sources, data types, stress inducers, ground truth, and validation methods. The difference in estimates between subgroups was considered statistically significant when the *P* value was <.05.

The Cochrane *Q* statistic was used to examine between-study heterogeneity, with a *P* value of <.05 indicating the presence of heterogeneity. The degree of between-study heterogeneity was evaluated using *I*^2^ [40,44], where it was deemed insignificant when *I*^2^ ranged from 0% to 40%, moderate when it ranged from 30% to 60%, substantial when it ranged from 50% to 90%, or considerable when it ranged from 75% to 100% [45].

## Results

### Search Results

**Figure 1:**
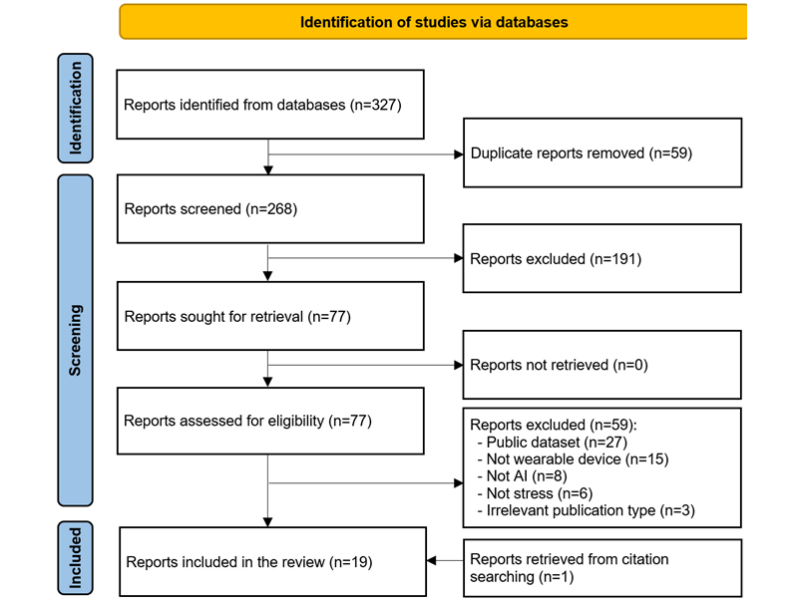
Flowchart of the study selection process. AI: artificial intelligence.

As shown in Figure 1, a total of 327 studies were retrieved when the databases identified previously were searched. Of the 327 retrieved studies, 59 (18%) duplicates were removed using EndNote X9, leaving 268 (82%) studies. Furthermore, 71.3% (191/268) of the studies were removed after we screened the titles and abstracts. After retrieving and reading the full texts of the remaining 77 studies, it was determined that 59 (77%) were ineligible for inclusion. The main reasons for exclusion were that they used a public data set, did not use wearable devices, did not use AI algorithms, did not focus on stress, or were irrelevant publication types. We identified an additional study relevant to this review through backward reference list checking. In total, 19 studies were included in this review [46-64], and 6 (32%) were eligible for the meta-analyses [46,52,53,55,62,63].

### Characteristics of the Included Studies

The key characteristics of the studies included in this review are presented in Table 1. The included studies were published between 2015 and 2023. The year in which the largest number of included studies was published was 2019 (5/19, 26%). The included studies were conducted in 10 distinct countries (Table 1), wherein the United States contributed to over a third of the studies (7/19, 37%). More than half (10/19, 53%) of the studies were conference papers, whereas the remaining studies were journal articles (8/19, 42%) and a preprint (1/19, 5%). The number of participants in the included studies ranged from 5 to 652, with an average of 77 (SD 149.5). The mean age of the participants was reported in 42% (8/19) of the included studies and ranged from 19.8 to 27.5 years, with an average of 22.8 (SD 2.5) years. A total of 58% (11/19) of the studies reported the proportion of female participants, which ranged from 14.3% to 76.8%, with an average of 47.3% (SD 21.5%). Participants in most of the studies (17/19, 89%) were undergraduate students. The characteristics of each included study are presented in Multimedia Appendix 5 [46-64].

**Table 1:** Characteristics of the included studies (N=19).

Feature			Values		References
**Year of publication, n (%)**					
	2023	2 (11)		[47,54]	
	2021	3 (16)		[46,53,63]	
	2020	4 (21)		[49,56,62,64]	
	2019	5 (26)		[48,52,55,57,58]	
	2018	3 (16)		[50,60,61]	
	2017	1 (5)		[51]	
	2015	1 (5)		[59]	
**Publication type, n (%)**					
	Conference paper	10 (53)		[46,51-53,55,57-59,61,64]	
	Journal article	8 (42)		[48-50,54,56,60,62,63]	
	Preprint	1 (5)		[47]	
**Country of publication, n (%)**					
	United States	7 (37)		[47,51,53-56,60]	
	India	3 (16)		[57,58,63]	
	United Kingdom	2 (11)		[49,59]	
	Others	1 (5) each		[46,48,50,52,61,62,64]	
Number of participants, mean (SD; range)			77 (149.5; 5-652)		[46-64]
**Age (y)**					
	Values, mean (SD; range)	22.76 (2.53; 19.8-27.5)		[47-49,54,56,61,62,64]	
	NR^a^, n (%)	11 (58)		[46,50-53,55,57-60,63]	
**Female participants**					
	Values (%), mean (SD; range)	47.3 (21.5; 14.3-76.8)		[47-49,52-54,56,60-62,64]	
	NR, n (%)	8 (42)		[46,50,51,55,57-59,63]	
**Educational level, n (%)**					
	High school	1 (5)		[48]	
	Undergraduate	17 (89)		[47-60,62-64]	
	Postgraduate	3 (16)		[49,61,63]	
	NR	1 (5)		[46]	

^a^NR: not reported.

### Features of the Wearable Devices

As depicted in Table 2, although 89% (17/19) of the studies used commercial wearable devices, 5% (1/19) used noncommercial wearable devices. The included studies used 16 different wearable devices. The wearable devices that were used by the largest number of studies were Empatica E4 (3/19, 16%) and Galaxy (Watch Active2 and Gear S, S2, and S3; 3/19, 16%). The most common type of wearable device used in the included studies was smart bands (12/19, 63%). The wearable devices were placed on 7 different parts of the body; however, the wrist was the most common (16/19, 84%). The periods for which the devices were worn in the included studies ranged from 10 minutes to 365 days. The features of the wearable devices in each included study are presented in Multimedia Appendix 6 [46-64].

**Table 2:** Features of the wearable devices (N=19).

Feature			Values		References
**Status of WD^a^, n (%)**					
	Commercial	17 (89)		[46-54,57-60,62-64]	
	Noncommercial	1 (5)		[51]	
	Not reported	2 (11)		[55,56]	
**Name of WD, n (%)**					
	Empatica E4	3 (16)		[48,61,64]	
	Galaxy (Watch Active2 and Gear S, S2, and S3)	3 (16)		[47,48,54]	
	Fitbit (Charge 2)	2 (11)		[53,58]	
	Microsoft Band 2	2 (11)		[50,62]	
	Others	1 (5) each		[46,49,51,52,54,59,60,63]	
	Not reported	2 (11)		[55,56]	
**Type of WD, n (%)**					
	Smart bands	12 (63)		[48-51,55,56,58-62,64]	
	Smartwatches	6 (32)		[46-48,51-54,63]	
	Electrodes	2 (11)		[52,63]	
	Others	1 (5) each		[54,57]	
**Placement of WD, n (%)**					
	Wrist	16 (84)		[46-51,53-56,58-62,64]	
	Chest	3 (16)		[51,52,63]	
	Finger	3 (16)		[54,59,63]	
	Palm	2 (11)		[52,63]	
	Others	1 (5) each		[57,63]	
**Duration of wearing WD**					
	Range	10 min to 365 d		N/A^b^	
	Not reported, n (%)	6 (32)		[46,50-52,57,62]	

^a^WD: wearable device.

^b^N/A: not applicable.

### Features of the AI

The AI algorithms in most of the included studies (18/19, 95%) were used to solve classification problems (Table 3). Most studies (15/19, 79%) used AI algorithms to categorize the sample into 2 classes (stressed vs not stressed). Among the included studies, 22 different algorithms were used, but the most commonly used algorithms were support vector machine (14/19, 74%) and k-nearest neighbor (13/19, 68%). All the included studies used AI to detect the current stress status, whereas none of them used AI to predict the occurrence of stress in the future. The data set size was reported in 47% (9/19) of the studies and ranged from 30 to 1178, with an average of 517.9 (SD 409). Data were collected using wearable devices (19/19, 100%), self-reported questionnaires (11/19, 58%), and nonwearable devices (eg, smartphones; 6/19, 32%). The included studies used 17 types of data to develop the models. However, the most common data types used to develop the models were HR data (eg, HR, HRV, and interbeat interval; 16/19, 84%), EDA data (10/19, 53%), and physical activity data (eg, step counts, calories, and metabolic rate) (8/19, 42%). The number of features extracted from the data varied between 4 and 498, with an average of 52.2 (SD 115.1). Stress inducers were used in 63% (12/19) of the studies, and the most common method used to induce stress among participants was arithmetic tasks (7/19, 37%). The ground truth was identified using self-reported questionnaires (eg, Perceived Stress Scale; 11/19, 58%), the context of the experiment (eg, stress exposure period and non–stress exposure period; 9/19, 47%), or unsupervised algorithms (1/19, 5%). The most common method used to validate the performance of the models was k-fold cross-validation (11/19, 58%). The most common measure used in the included studies to evaluate the performance of AI algorithms was accuracy (16/19, 84%). The features of the AI in each included study are described in Multimedia Appendix 7 [46-64].

**Table 3:** Features of the artificial intelligence (AI; N=19).

Feature		Values	References
**Problem-solving approaches, n (%)**			
	Classification	18 (95)	[46-63]
	Regression	1 (5)	[50]
	Clustering	1 (5)	[64]
**Number of stress classes, n (%)**			
	2	15 (79)	[46,47,49,51,53-62,64]
	3	5 (26)	[48,50,52,57,61]
	>3	2 (11)	[55,63]
**AI algorithms, n (%)**			
	Support vector machine	14 (74)	[46,48,50-57,59,60,62,63]
	K-nearest neighbor	13 (68)	[46,47,50,52-57,61-63]
	Random forest	8 (42)	[47,50,51,54,55,61-63]
	Logistic regression	6 (32)	[46,48,51,53,62,63]
	Decision tree	5 (26)	[50,52,55,58,61]
	Naïve Bayes	5 (26)	[46,50,51,58,62]
	Artificial neural network	4 (21)	[53,55,62,63]
	Multilayer perceptron	4 (21)	[46,48,54,56]
	Others	<4	[46-50,52,55,56,60-64]
**Aim of AI algorithms, n (%)**			
	Detection	19 (100)	[46-64]
Data set size, mean (SD; range)		518 (409; 30-1178)	[46,47,52,53,55,61-64]
**Data sources, n (%)**			
	Wearable devices	19 (100)	[46-64]
	Self-reported questionnaire	11 (58)	[47,49,51,53-55,58,60,62,63]
	Nonwearable devices	6 (32)	[47,53,54,58,60,61]
**Data types, n (%)**			
	Heart rate data	16 (84)	[46-54,56,58,59,61,63,64]
	Electrodermal activity data	10 (53)	[48,50-52,54,59-61,63,64]
	Activity data	8 (42)	[47,48,53-55,58,60,61]
	Sleep data	5 (26)	[53-55,58,60]
	Skin temperature	3 (16)	[48,50,60]
	Smartphone use data	3 (16)	[47,54,60]
	Location	3 (16)	[47,54,60]
	Respiratory rate data	3 (16)	[47,54,61]
	Others	<3	[47,53,54,57,58,60,61]
**Number of features**			
	Values, mean (SD; range)	52.2 (115.1; 4-498)	[46-64]
	Not reported, n (%)	1 (5)	[55]
**Stress-induction methods, n (%)**			
	Arithmetic tasks	7 (37)	[46,50,51,56,59,61,63]
	Exams	4 (21)	[49,50,52,62]
	Stroop test	4 (21)	[50,51,56,63]
	Physical stress	3 (16)	[50,51,56]
	Others	<3	[48,50-52,56,59,61,64]
	No stress induction	7 (37)	[47,53-55,57,58,60]
**Ground truth, n (%)**			
	Self-reported questionnaire	11 (58)	[47-49,51,53-55,57,58,60,62]
	Context	9 (47)	[48,50-52,56,59,61,63,64]
	Unsupervised algorithm	1 (5)	[46]
**Types of validation, n (%)**			
	K-fold	11 (58)	[46-48,50-54,56,57,63]
	Training-test split	6 (32)	[46,49,55,57,59,62]
	Leave-one-out cross-validation	2 (11)	[51,61]
	Nested	1 (5)	[60]
	Not reported	1 (5)	[58]
	Not applicable	1 (5)	[64]
**Performance measures, n (%)**			
	Accuracy	16 (84)	[46,48-50,52-61,63,64]
	*F*_1_-score	9 (47)	[46-48,51,55,56,60,61,64]
	Precision	7 (37)	[48,51,55,56,58,59,64]
	Sensitivity	6 (32)	[48,55,56,58,62,64]
	κ	3 (16)	[46,50,51]
	Others	<4	[46,50,51,58,62,64]

### Results of the Risk-of-Bias Appraisal

**Figure 2:**
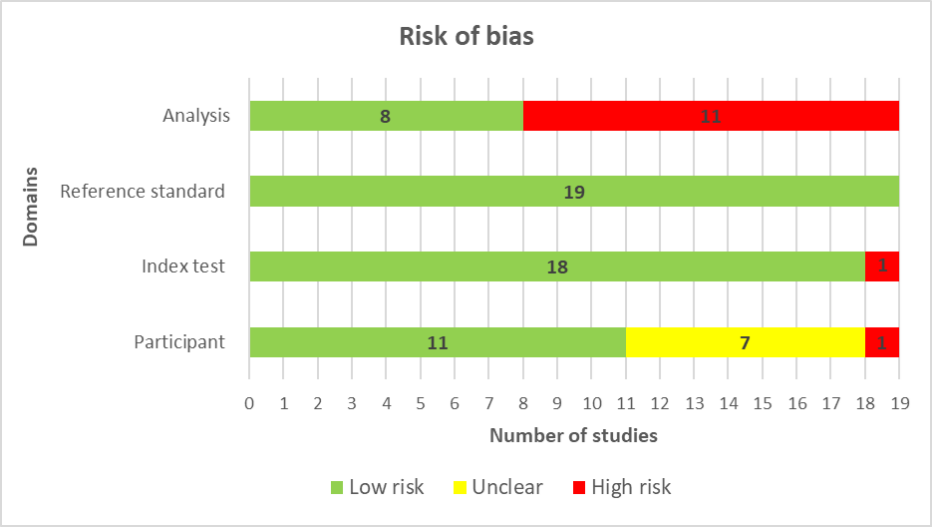
Results of the assessment of risk of bias in the included studies.

All studies (19/19, 100%) reported comprehensive details to determine whether an appropriate consecutive or random sample of eligible patients was used, and none of the studies had inappropriate exclusions. Nearly half (8/19, 42%) of the studies ensured a balanced number of patients across subgroups. Only a third (6/19, 32%) reported a sufficient sample size, leaving ambiguity regarding the adequacy of the sample size in most of the studies (13/19, 68%). As a result, only a little over half (11/19, 58%) of the studies were assessed as having a low risk of bias from the “selection of participants” domain (Figure 2). In terms of matching participants to the predefined requirements in the review question, a low level of concern was identified in most of the studies, accounting for 68% (13/19).

**Figure 3:**
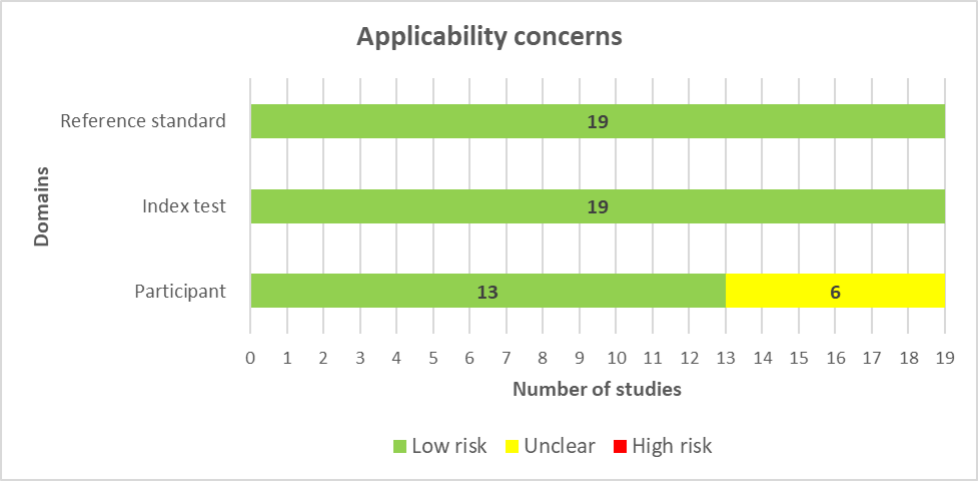
Results of the assessment of applicability concerns in the included studies.

All studies in our review (19/19, 100%) comprehensively detailed the AI models, clearly reported the features (predictors) used, and ensured that these features were sourced without previous knowledge of the outcome data. For nearly every study (18/19, 95%), the features were consistently assessed across participants. Therefore, for the vast majority of the studies (18/19, 95%), the potential for bias in the “index test” was assessed as low (Figure 2). Consistently, all studies (19/19, 100%) were found to have minimal concerns regarding the match between the model’s predictors and the review question’s criteria (Figure 3).

In all the reviewed studies (19/19, 100%), the outcome of interest, specifically the stress level, was consistently assessed using appropriate methodologies. Similarly, outcomes were defined uniformly for participants across studies and determined without access to predictor data, and an appropriate time gap was ensured between the index test and the reference standard. As a result, the potential for bias regarding the “reference standard” domain was deemed low for all studies (19/19, 100%; Figure 2). In addition, all studies (19/19, 100%) showed minimal concerns over discrepancies between the outcome’s definition, timing, or determination and the review question’s criteria (Figure 3).

Finally, two-thirds of the studies (12/19, 63%) ensured that all the enrolled participants were factored into the data analysis. Over half (10/19, 53%) of these studies executed proper data preprocessing, whereas in 68% (13/19), there was an appropriate split between training, validation, and test sets. The same proportion of studies (13/19, 68%) adopted suitable measures to evaluate the performance of their models. However, considering all these factors, only a minority (8/19, 42%) were deemed to have a minimal “analysis” domain risk of bias (Figure 2). A detailed breakdown of the “risk of bias” and “applicability concerns” for every domain in each study is available in Multimedia Appendix 8 [46-64].

### Results of the Studies

#### Overview

Accuracy, sensitivity, specificity, and *F*_1_-score were reported in 89% (17/19), 32% (6/19), 11% (2/19), and 53% (10/19) of the studies, respectively. Accuracy values were synthesized using both narrative and statistical methods, whereas the values of the remaining measures were synthesized solely through narrative methods. This distinction arises from the fact that 35% (6/17) of the studies that assessed accuracy reported enough data to conduct meta-analyses, whereas none of the studies evaluating the other measures had sufficient data for such analyses. The results related to accuracy and other measures are presented in the following subsections.

#### Accuracy

Wearable AI accuracy was assessed in 89% (17/19) of the studies. From these studies, we extracted the highest accuracy for each algorithm in each study; therefore, we identified 71 accuracy estimates. The estimates of accuracy ranged from 0.48 to 1.00, with an average of 0.822 (SD 0.135). We conducted meta-analyses of 37 estimates derived from 11,425 participants across 35% (6/17) of the studies (Table 4). The pooled mean accuracy of these estimates was 0.856 (95% CI 0.698-0.934). The meta-analyzed evidence exhibited considerable statistical heterogeneity (*P*<.001; *I*^2^=98.8%). Furthermore, subgroup meta-analyses were conducted to assess the performance of wearable AI based on different factors. As shown in Table 4, there was a statistically significant difference in pooled accuracy between subgroups in the number of stress classes group (*P*=.02), type of wearable device group (*P*=.049), location of wearable device group (*P*=.02), data set size group (*P*=.009), and ground truth group (*P*=.001), whereas no statistically significant difference was found in the pooled accuracy between subgroups in the remaining groups.

**Table 4:** Pooled mean estimates of accuracy by several factors (N=19).

Group			Number of studies^a^		Sample size, n		Accuracy (%), range		Pooled mean accuracy (%), mean (95% CI)		Heterogeneity measures							Test for subgroup differences (*P* value)	
											τ^2^		Q (*P* value)		*I*^2^ (%)				
**Algorithm**																			.98
	Support vector machine	6		2039		0.65-0.94		0.796 (0.67-0.90)		0.0276		143.0 (<.001)		96.5					
	K-nearest neighbor	6		2039		0.58-0.98		0.834 (0.71-0.93)		0.0326		96.2 (<.001)		94.8					
	Logistic regression	4		1545		0.66-0.87		0.768 (0.67-0.86)		0.0108		20.7 (<.001)		85.5					
	Artificial neural network	4		1686		0.66-0.99		0.811 (0.63-0.94)		0.0436		58.3 (<.001)		94.9					
	Random forest	3		1215		0.71-0.98		0.829 (0.61-0.97)		0.0456		43.1 (<.001)		95.4					
	Decision tree	2		494		0.64-0.86		0.762 (0.52-0.94)		0.0319		29.6 (<.001)		96.6					
	Naïve Bayes	2		990		0.68-0.97		0.841 (0.50-1.00)		0.0672		16.9 (<.001)		94.1					
**Number of stress classes**																			*.02^b^*
	2	25		9222		0.58-0.97		0.773 (0.61-0.87)		0.0873		173.4 (<.001)		97.2					
	>2	12		2203		0.86-0.99		0.944 (0.77-0.99)		0.2110		302.8 (<.001)		98.2					
**Type of WD^c^**																			*.049*
	Smartwatches	11		2094		0.66-0.97		0.836 (0.53-0.95)		0.1466		91.6 (<.001)		96.6					
	Smart bands	14		7128		0.58-0.77		0.695 (0.63-0.75)		0.0050		69.2 (<.001)		85.4					
	Electrodes	12		2203		0.86-0.99		0.944 (0.77-0.99)		0.2110		302.8 (<.001)		98.2					
**Location of WD**																			*.02*
	Wrist	25		9222		0.58-0.97		0.773 (0.61-0.87)		0.0873		173.4 (<.001)		97.2					
	Nonwrist	12		2203		0.86-0.99		0.944 (0.77-0.99)		0.2110		302.8 (<.001)		98.2					
**Data set size**																			*.009*
	≤100	14		789		0.80-0.99		0.947 (0.81-0.99)		0.1704		166.7 (<.001)		94.1					
	>100	23		10,627		0.58-0.91		0.769 (0.62-0.86)		0.0758		485.3 (<.001)		97.4					
**Data source**																			.57
	WD-based	12		1825		0.80-0.97		0.895 (0.87-0.91)		0.0000		29.9 (<.001)		60.5					
	WD-based data+other data	25		9600		0.58-0.99		0.830 (0.50-0.95)		0.3708		968.9 (<.001)		99.4					
**Data type**																			.21
	Heart rate data	13		5970		0.65-0.97		0.832 (0.52-0.95)		0.1594		115.4 (<.001)		98.6					
	Heart rate data+EDA^d^ data	12		2203		0.86-0.99		0.944 (0.77-0.99)		0.2110		302.8 (<.001)		98.2					
**Stress inducers**																			.10
	Yes	25		8173		0.65-0.99		0.902 (0.75-0.96)		0.2292		1137.8 (<.001)		98.9					
	No	12		3252		0.58-0.80		0.697 (0.62-0.76)		0.0060		51.4 (<.001)		80.6					
**Ground truth**																			*.001*
	Objective	19		2413		0.80-0.99		0.933 (0.84-0.97)		0.1266		320.2 (<.001)		96.3					
	Subjective	18		9012		0.58-0.80		0.706 (0.66-0.75)		0.0031		95.0 (<.001)		85.1					
**Validation method**																			.16
	K-fold	16		4087		0.66-0.99		0.904 (0.64-0.98)		0.3324		784.7 (<.001)		99.0					
	Training-test split	14		7128		0.58-0.77		0.695 (0.63-0.75)		0.0050		69.2 (<.001)		85.4					
All studies			37		11,425		0.58-0.99		0.856 (0.698-0.934)		0.2402		1318.2 (<.001)		98.8		N/A^e^		

^a^Many studies were included more than once in all meta-analyses except for the meta-analysis related to algorithms given that the studies assessed the performance of more than one algorithm.

^b^Italics indicate statistical significance (*P*<.05).

^c^WD: wearable device.

^d^EDA: electrodermal activity.

^e^N/A: not applicable.

#### Other Measures

The sensitivity of wearable AI in detecting stress was reported in 32% (6/19) of the studies. From these studies, 27 sensitivity estimates were identified given that we extracted the highest sensitivity for each algorithm in each study. The estimates of sensitivity varied between 0.42 and 0.97, with an average of 0.755 (SD 0.181). From the 11% (2/19) of studies that reported specificity, 8 estimates of specificity were extracted given that more than one algorithm was assessed in these studies. The estimates of specificity ranged between 0.475 and 0.95, with an average of 0.744 (SD 0.147). We identified 46 estimates of *F*_1_-score from 53% (10/19) of the studies. The estimates of the *F*_1_-score ranged from 0.514 to 0.979, with an average of 0.759 (SD 0.139).

## Discussion

### Principal Findings

This review assessed the performance of wearable AI in detecting stress among students. Our analyses showed that wearable AI has an acceptable performance in detecting stress among students, but there is still room for improvement. Specifically, our meta-analyses revealed that wearable AI was able to correctly classify students with and without stress in 85.6% of cases. Furthermore, our narrative synthesis showed that the performance of wearable AI in detecting students with stress (75.5%) is comparable with its performance in detecting students without stress (74.4%). We considered that the performance of wearable AI in detecting stress among students is suboptimal given that many previous reviews have shown a higher performance of AI in detecting other diseases or disorders, such as cancers [65-68], heart diseases [69,70], ear diseases [71], and ophthalmic disorders [72,73]. Moreover, relying solely on pooled mean accuracy is insufficient for drawing definitive conclusions regarding the performance of wearable AI; thus, we considered sensitivity and specificity when evaluating its performance, which were lower than the pooled accuracy.

The subgroup analyses in this review demonstrated that the performance of wearable AI is moderated by 5 factors. The first factor is the type of wearable device used to collect digital biomarkers. To be more precise, electrodes have higher accuracy (94.4%) in detecting stress than smartwatches (83.6%) and smart bands (69.5%). This can be explained by the practice of typically positioning electrodes on the most suitable areas of the body for gathering specific biomarkers, thereby collecting more accurate data, as opposed to most commercial and fashionable popular devices being restricted to wrists.

The second moderator identified in this review is the placement of wearable devices on the body. To elaborate, studies that used wrist-worn devices exhibited lower accuracy in detecting stress among students compared with those that used wearable devices placed on other parts of the body (94.4% vs 77.3%). This can be attributed to 4 factors. First, all studies that used non–wrist-worn devices collected data from more than one area of the body (eg, chest, fingers, and palm) and used electrodes, whereas all studies that used wrist-worn devices collected data from only one area (ie, wrist) and used smartwatches or smart bands. Second, the placement of wearable devices on the wrist may lead to less accurate data collection because of the frequent arm movements. Third, all studies that used non–wrist-worn devices collected EDA data, whereas none of the studies that used wrist-worn devices collected EDA data. Finally, the ground truth in all studies that used non–wrist-worn devices was assessed objectively, whereas the ground truth in approximately half of the studies that used wrist-worn devices was identified subjectively.

The third moderator identified in this review is the number of stress classes that wearable AI endeavors to detect. Surprisingly, our analysis revealed that wearable AI exhibits greater accuracy in classifying more than 2 stress levels (94.4%) as opposed to just 2 stress classes (77.3%). This may be attributed to the same 4 aforementioned factors given that the estimates used in the subgroup meta-analysis to assess the performance of wearable AI in classifying more than 2 stress levels were the exact estimates used in the meta-analysis to evaluate the performance of non–wrist-worn devices.

The fourth moderator found in this review is the ground truth. Specifically, the efficacy of wearable AI in stress detection tends to be superior when the ground truth is determined through objective assessments (eg, the experimental context), in contrast to when it relies on subjective assessments (ie, self-reported questionnaires; 93.3% vs 70.6%). This is expected as objective assessments are generally more reliable and accurate than subjective assessments.

The last moderator identified in this review is the data set size. Unexpectedly, our subgroup meta-analyses demonstrated that the accuracy of wearable AI in detecting stress is higher when using a data set size of ≤100 (94.7%) as opposed to data sets with sizes of >100 (76.9%). Although this seems odd at first glance, the reason could be that the ground truth in all studies that used a data set size of ≤100 was examined objectively, whereas the ground truth in approximately half of the studies that used a data set size of >100 was identified subjectively.

As mentioned in the *Introduction* section, none of the previous reviews statistically synthesized the results of previous studies. Thus, we calculated the traditional average of accuracy estimates reported in the studies included in these reviews. We noticed comparable findings despite the differences between our review and these reviews. To elaborate, although the pooled mean in this review was 85.6%, the average accuracy estimates in the previous reviews were 87.6% (50%-100%) [28], 87% (70.8%-99%) [34], 86% (60%-100%) [15], 85.4% (64.5%-98.3%) [35], 84.2% (51.2%-100%) [24], and 82.9% (53%-99%) [13]. Furthermore, this review showed findings comparable with those of previous studies on the performance of wearable AI in detecting depression [74] and anxiety [75]. Specifically, the pooled mean accuracies of wearable AI in detecting depression and anxiety were 89% [74] and 82% [75], respectively.

### Research and Practical Implications

This review found that wearable AI holds promise as a valuable tool for detecting stress among students. Nonetheless, we are unable to endorse the immediate integration of wearable AI into clinical and academic practices for the following reasons: (1) its efficiency in detecting stress among students is currently suboptimal, (2) the number of participants was small (≤50) in 74% (14/19) of the studies, (3) the accuracy estimates included in all meta-analyses were extracted from a few studies (≤6), and (4) only 21% (4/19) of the studies were judged to have a low risk of bias in all domains. Hence, it is advisable to use wearable AI in conjunction with other clinical assessments such as self-report questionnaires to detect stress among students.

Most of the studies included in this review (17/19, 89%) targeted undergraduate students, although students at other educational levels are vulnerable to stress. Hence, future research endeavors should recruit students at different educational levels, such as postgraduate and high school students. This review primarily focused on stress among students even though there is a substantial body of research on the effectiveness of wearable AI in identifying stress in other demographic groups. Thus, further systematic reviews are needed to consolidate and synthesize the evidence on the performance of wearable AI in detecting stress across diverse populations.

In this review, all the included studies evaluated the effectiveness of wearable AI in detecting current stress levels rather than predicting the occurrence of stress in the future. Anticipating the onset of stress in the future is equally if not more crucial than identifying the current stress state as this can facilitate the development and implementation of more efficient, timely, and tailored interventions. For this reason, we urge researchers to conduct further studies on the potential of wearable AI for predicting future occurrences of stress.

None of the studies included in this review evaluated the effectiveness of wearable AI in differentiating stress from other mental health conditions such as depression and anxiety, nor did they assess its ability to distinguish between different types of stress (eg, acute stress, chronic stress, emotional stress, physical stress, eustress, distress, and posttraumatic stress disorder). In clinical practice, health care professionals often rely on complex and error-prone diagnostic methods to make distinctions among various patient groups rather than merely distinguishing them from individuals without health issues. Consequently, it is imperative for researchers to investigate how well wearable AI can differentiate between different types of stress and distinguish individuals with stress from those who have other mental disorders exhibiting signs and symptoms similar to those of stress.

Although previous studies have examined the performance of wearable AI based on different factors (eg, algorithms, data types, and number of stress classes), they did not assess its performance based on the placement of the wearable device (eg, wrist, chest, or fingers), the methods used to induce stress (eg, Stroop test, arithmetic tasks, and physical exercises), or the methods used to assess the ground truth (eg, subjective and objective assessments). We strongly encourage researchers to consider these factors in future studies. Furthermore, none of the previous studies have used biological samples as the ground truth, although they are considered as the gold-standard test of stress. For example, elevated cortisol levels in blood, saliva, or urine samples can be indicative of chronic stress. Such gold-standard tests, especially those that can be measured easily and noninvasively, should be considered in future studies.

Even though this review included 19 studies, we were able to include results from only 6 (32%) in our meta-analyses as the remaining studies did not provide sufficient details essential for conducting meta-analyses (eg, confusion matrices, number of cases, and number of controls). In addition, these studies did not present multiple performance measures such as accuracy, sensitivity, specificity, and *F*_1_-score, which are necessary for us to estimate the required details. Hence, researchers should include the aforementioned details in their reports to facilitate the conduct of meta-analyses by others.

A total of 74% (14/19) of the studies had a limited number of participants, with ≤50 individuals involved. This could have posed challenges in identifying potential performance differences in wearable AI when conducting subgroup meta-analyses. In addition, this could have acted as a hindrance for the development of algorithms that rely on a substantial amount of data. To address these issues, it is essential for researchers to recruit a larger number of participants, ensuring robust statistical power and enabling the use of more advanced and efficient algorithms.

### Limitations

This review excluded studies that assessed the performance of (1) nonwearable devices, handheld devices, near-body wearable devices, in-body wearable devices, wearable devices wired to nonwearable devices, and wearables requiring expert oversight; (2) wearable AI in detecting other mental disorders (eg, attention-deficit/hyperactivity disorder, bipolar disorder, and schizophrenia); (3) wearable AI in detecting stress among populations other than students; and (4) wearable AI in predicting outcomes of stress treatment. Consequently, the generalizability of our findings to such devices, disorders, populations, and outcomes is limited.

This review could not draw definitive conclusions based on the results of the meta-analyses for several reasons. First, it is probable that we overlooked some studies as we excluded studies that were published in non-English languages and before 2015 and used publicly available data sets (eg, WESAD) or data sets from previous studies. Second, the accuracy estimates included in all meta-analyses were drawn from a limited number of studies (≤6), primarily as the remaining studies lacked the necessary information required for conducting meta-analyses.

### Conclusions

Wearable AI holds promise as a valuable tool for detecting stress among students. However, it is not sufficiently ready to be integrated into clinical and academic practices given its suboptimal performance. Until further evidence demonstrates an ideal performance of wearable AI, it should be used in conjunction with other clinical assessments to help detect stress among students. Given the limitations of the current evidence, researchers should investigate how well wearable AI can differentiate between different types of stress and distinguish individuals with stress from those who have other mental disorders exhibiting signs and symptoms similar to those of stress. Furthermore, future research should recruit students at different educational levels, such as postgraduate and high school students. There is also a need to conduct studies on the potential of wearable AI for predicting future occurrences of stress. Future studies should compare the performance of wearable AI based on the placement of the wearable device, the methods used to induce stress, and the methods used to assess the ground truth. Finally, researchers should provide detailed results to facilitate the conduct of meta-analyses by others.

## Appendix

Multimedia Appendix 1 PRISMA-DTA (Preferred Reporting Items for Systematic Reviews and Meta-Analyses extension for Diagnostic Test Accuracy) checklist.

Multimedia Appendix 2 Search strategy.

Multimedia Appendix 3 Data extraction form.

Multimedia Appendix 4 Modified version of the Quality Assessment of Diagnostic Accuracy Studies–Revised.

Multimedia Appendix 5 Characteristics of each included study.

Multimedia Appendix 6 Features of wearable devices.

Multimedia Appendix 7 Features of the artificial intelligence algorithms.

Multimedia Appendix 8 Reviewers’ judgments about each risk of bias and applicability domain for each included study.

## Abbreviations

AI: artificial intelligence
EDA: electrodermal activity
HR: heart rate
HRV: heart rate variability
PRISMA-DTA: Preferred Reporting Items for Systematic Reviews and Meta-Analyses extension for Diagnostic Test Accuracy
QUADAS-2: Quality Assessment of Diagnostic Accuracy Studies–Revised
WESAD: Wearable Stress and Affect Detection

## Data Availability

The data that support the findings of this study are available from the corresponding author upon reasonable request.
