# Supplementary material for: The Performance of Wearable AI in Detecting Stress Among Students: Systematic Review and Meta-Analysis
Source: J Med Internet Res. 2024 Jan 31;26:e52622. doi: 10.2196/52622 (PMC10867751; doi:10.2196/52622)
Supplement: Multimedia Appendix 2 [file jmir_v26i1e52622_app2.docx]

| Monday, May 30, 2022 6:45:26 AM Monday, May 30, 2022 6:39:40 AM |
| --- |

**Multimedia Appendix 2: Search Strategy**

Database(s): **Ovid MEDLINE(R) ALL**1946 to June 12, 2023
Search Strategy:

| **#** | **Searches** | **Results** |
| --- | --- | --- |
| 1 | exp Artificial Intelligence/ | 173497 |
| 2 | Artificial Intelligence.tw. | 29381 |
| 3 | exp Machine Learning/ | 57538 |
| 4 | "Machine Learning".tw. | 77415 |
| 5 | exp Deep Learning/ | 15649 |
| 6 | "Deep Learning".tw. | 41216 |
| 7 | "Supervised learning".tw. | 4684 |
| 8 | "unsupervised learning".tw. | 2095 |
| 9 | "reinforcement learning".tw. | 5299 |
| 10 | "Computer vision".tw. | 6904 |
| 11 | "Decision tree*".tw. | 14295 |
| 12 | "K-Nearest Neighbor*".tw. | 4840 |
| 13 | "Support vector machine*".tw. | 24562 |
| 14 | "convolutional neural network*".tw. | 21608 |
| 15 | "Recurrent neural network*".tw. | 3958 |
| 16 | "Artificial neural network*".tw. | 16530 |
| 17 | "deep neural network*".tw. | 8372 |
| 18 | "Naïve Bayes".tw. | 7 |
| 19 | "Naive Bayes".tw. | 3023 |
| 20 | "Fuzzy Logic".tw. | 2366 |
| 21 | "logistic regression".tw. | 392313 |
| 22 | "K-Means".tw. | 6783 |
| 23 | "Random Forest".tw. | 18576 |
| 24 | "Long Short-Term Memory Networks".tw. | 175 |
| 25 | "Gradient Boosting".tw. | 4233 |
| 26 | "U-Net".tw. | 3352 |
| 27 | "Capsule Network*".tw. | 210 |
| 28 | "Autoencoder*".tw. | 2729 |
| 29 | "boltzmann machine".tw. | 300 |
| 30 | "deep belief network".tw. | 305 |
| 31 | "Gradient Boost*".tw. | 4951 |
| 32 | AdaBoost.tw. | 1264 |
| 33 | "Multilayer Perceptron".tw. | 2455 |
| 34 | exp Wearable Electronic Devices/ | 18760 |
| 35 | wearable*.tw. | 24241 |
| 36 | "smart watch*".tw. | 213 |
| 37 | smartwatch*.tw. | 939 |
| 38 | acceleromet*.tw. | 21673 |
| 39 | gyroscop*.tw. | 2401 |
| 40 | "inertial sensor".tw. | 1123 |
| 41 | "inertial measurement unit*".tw. | 2721 |
| 42 | "fitness band*".tw. | 24 |
| 43 | headband*.tw. | 352 |
| 44 | "head band*".tw. | 74 |
| 45 | wristband*.tw. | 743 |
| 46 | "wrist band*".tw. | 98 |
| 47 | "smart insole*".tw. | 50 |
| 48 | bracelet*.tw. | 667 |
| 49 | Emotiv.tw. | 90 |
| 50 | NeuroSky.tw. | 18 |
| 51 | Mindo.tw. | 44 |
| 52 | Muse.tw. | 644 |
| 53 | Fitbit.tw. | 1151 |
| 54 | Garmin.tw. | 257 |
| 55 | "Polar loop".tw. | 32 |
| 56 | Jawbone.tw. | 894 |
| 57 | Geneactiv.tw. | 182 |
| 58 | Empatica.tw. | 91 |
| 59 | Actigraph.tw. | 3532 |
| 60 | "Apple Watch".tw. | 271 |
| 61 | Amazfit.tw. | 5 |
| 62 | Actiwatch.tw. | 379 |
| 63 | "Mi Band".tw. | 43 |
| 64 | "Oura Ring".tw. | 33 |
| 65 | Vivosmart.tw. | 40 |
| 66 | "Microsoft Band".tw. | 22 |
| 67 | "Actiwave cardio".tw. | 3 |
| 68 | "MindWave Mobile".tw. | 3 |
| 69 | "Galaxy watch".tw. | 13 |
| 70 | Biobeam.tw. | 5 |
| 71 | Stress, Psychological/ | 133502 |
| 72 | stress*.tw. | 1057932 |
| 73 | distress.tw. | 148981 |
| 74 | Students/ | 79804 |
| 75 | Student*.tw. | 357403 |
| 76 | Pupils.tw. | 10962 |
| 77 | Graduate*.tw. | 56325 |
| 78 | Undergraduate*.tw. | 52110 |
| 79 | Postgraduate*.tw. | 20350 |
| 80 | Collegian*.tw. | 43 |
| 81 | Sophomore*.tw. | 866 |
| 82 | schoolchildren.tw. | 15392 |
| 83 | 1 or 2 or 3 or 4 or 5 or 6 or 7 or 8 or 9 or 10 or 11 or 12 or 13 or 14 or 15 or 16 or 17 or 18 or 19 or 20 or 21 or 22 or 23 or 24 or 25 or 26 or 27 or 28 or 29 or 30 or 31 or 32 or 33 | 678375 |
| 84 | 34 or 35 or 36 or 37 or 38 or 39 or 40 or 41 or 42 or 43 or 44 or 45 or 46 or 47 or 48 or 49 or 50 or 51 or 52 or 53 or 54 or 55 or 56 or 57 or 58 or 59 or 60 or 61 or 62 or 63 or 64 or 65 or 66 or 67 or 68 or 69 or 70 | 65650 |
| 85 | 71 or 72 or 73 | 1223642 |
| 86 | 74 or 75 or 76 or 77 or 78 or 79 or 80 or 81 or 82 | 458634 |
| 87 | 83 and 84 and 85 and 86 | 54 |
| 88 | limit 87 to (english language and yr="2015 -Current") | 52 |

Database(s): **Embase**1974 to 2023 Week 23
Search Strategy:

| **#** | **Searches** | **Results** |
| --- | --- | --- |
| 1 | exp Artificial Intelligence/ | 82368 |
| 2 | Artificial Intelligence.tw. | 37296 |
| 3 | exp Machine Learning/ | 407497 |
| 4 | "Machine Learning".tw. | 95950 |
| 5 | exp Deep Learning/ | 43073 |
| 6 | "Deep Learning".tw. | 50429 |
| 7 | "Supervised learning".tw. | 5383 |
| 8 | "unsupervised learning".tw. | 2393 |
| 9 | "reinforcement learning".tw. | 6132 |
| 10 | "Computer vision".tw. | 7572 |
| 11 | "Decision tree*".tw. | 20535 |
| 12 | "K-Nearest Neighbor*".tw. | 5792 |
| 13 | "Support vector machine*".tw. | 30133 |
| 14 | "convolutional neural network*".tw. | 26622 |
| 15 | "Recurrent neural network*".tw. | 4572 |
| 16 | "Artificial neural network*".tw. | 19611 |
| 17 | "deep neural network*".tw. | 9798 |
| 18 | "Naïve Bayes".tw. | 23 |
| 19 | "Naive Bayes".tw. | 3776 |
| 20 | "Fuzzy Logic".tw. | 2892 |
| 21 | "logistic regression".tw. | 583467 |
| 22 | "K-Means".tw. | 9247 |
| 23 | "Random Forest".tw. | 23898 |
| 24 | "Long Short-Term Memory Networks".tw. | 200 |
| 25 | "Gradient Boosting".tw. | 5188 |
| 26 | "U-Net".tw. | 4674 |
| 27 | "Capsule Network*".tw. | 222 |
| 28 | "Autoencoder*".tw. | 3103 |
| 29 | "boltzmann machine".tw. | 340 |
| 30 | "deep belief network".tw. | 371 |
| 31 | "Gradient Boost*".tw. | 6266 |
| 32 | AdaBoost.tw. | 1590 |
| 33 | "Multilayer Perceptron".tw. | 2792 |
| 34 | exp Wearable Electronic Devices/ | 8491 |
| 35 | wearable*.tw. | 27232 |
| 36 | "smart watch*".tw. | 331 |
| 37 | smartwatch*.tw. | 1187 |
| 38 | acceleromet*.tw. | 27645 |
| 39 | gyroscop*.tw. | 2417 |
| 40 | "inertial sensor".tw. | 1332 |
| 41 | "inertial measurement unit*".tw. | 2962 |
| 42 | "fitness band*".tw. | 32 |
| 43 | headband*.tw. | 523 |
| 44 | "head band*".tw. | 112 |
| 45 | wristband*.tw. | 1083 |
| 46 | "wrist band*".tw. | 181 |
| 47 | "smart insole*".tw. | 53 |
| 48 | bracelet*.tw. | 1008 |
| 49 | Emotiv.tw. | 140 |
| 50 | NeuroSky.tw. | 22 |
| 51 | Mindo.tw. | 54 |
| 52 | Muse.tw. | 1285 |
| 53 | Fitbit.tw. | 1796 |
| 54 | Garmin.tw. | 362 |
| 55 | "Polar loop".tw. | 39 |
| 56 | Jawbone.tw. | 993 |
| 57 | Geneactiv.tw. | 266 |
| 58 | Empatica.tw. | 118 |
| 59 | Actigraph.tw. | 5398 |
| 60 | "Apple Watch".tw. | 433 |
| 61 | Amazfit.tw. | 7 |
| 62 | Actiwatch.tw. | 990 |
| 63 | "Mi Band".tw. | 55 |
| 64 | "Oura Ring".tw. | 55 |
| 65 | Vivosmart.tw. | 47 |
| 66 | "Microsoft Band".tw. | 26 |
| 67 | "Actiwave cardio".tw. | 4 |
| 68 | "MindWave Mobile".tw. | 5 |
| 69 | "Galaxy watch".tw. | 23 |
| 70 | Biobeam.tw. | 8 |
| 71 | Stress, Psychological/ | 86403 |
| 72 | stress*.tw. | 1328226 |
| 73 | distress.tw. | 210572 |
| 74 | Students/ | 105189 |
| 75 | Student*.tw. | 464658 |
| 76 | Pupils.tw. | 15500 |
| 77 | Graduate*.tw. | 71050 |
| 78 | Undergraduate*.tw. | 61384 |
| 79 | Postgraduate*.tw. | 27583 |
| 80 | Collegian*.tw. | 51 |
| 81 | Sophomore*.tw. | 1023 |
| 82 | schoolchildren.tw. | 17941 |
| 83 | 1 or 2 or 3 or 4 or 5 or 6 or 7 or 8 or 9 or 10 or 11 or 12 or 13 or 14 or 15 or 16 or 17 or 18 or 19 or 20 or 21 or 22 or 23 or 24 or 25 or 26 or 27 or 28 or 29 or 30 or 31 or 32 or 33 | 1065065 |
| 84 | 34 or 35 or 36 or 37 or 38 or 39 or 40 or 41 or 42 or 43 or 44 or 45 or 46 or 47 or 48 or 49 or 50 or 51 or 52 or 53 or 54 or 55 or 56 or 57 or 58 or 59 or 60 or 61 or 62 or 63 or 64 or 65 or 66 or 67 or 68 or 69 or 70 | 71675 |
| 85 | 71 or 72 or 73 | 1535585 |
| 86 | 74 or 75 or 76 or 77 or 78 or 79 or 80 or 81 or 82 | 597616 |
| 87 | 83 and 84 and 85 and 86 | 68 |
| 88 | limit 87 to (english language and yr="2015 -Current") | 61 |

Database(s): **APA PsycInfo**2002 to June Week 2 2023. Tuesday, June 13, 2023

| **#** | **Searches** | **Results** |
| --- | --- | --- |
| 1 | exp Artificial Intelligence/ | 23666 |
| 2 | "Artificial Intelligence".tw. | 4908 |
| 3 | exp Machine Learning/ | 12207 |
| 4 | "Machine Learning".tw. | 9527 |
| 5 | exp Deep Learning/ | 0 |
| 6 | "Deep Learning".tw. | 2575 |
| 7 | "supervised learning".tw. | 987 |
| 8 | "unsupervised learning".tw. | 558 |
| 9 | "reinforcement learning".tw. | 2981 |
| 10 | "Decision tree".tw. | 1296 |
| 11 | "K-Nearest Neighbor*".tw. | 443 |
| 12 | "Support vector machine*".tw. | 3057 |
| 13 | "Recurrent neural network*".tw. | 911 |
| 14 | "convolutional neural network*".tw. | 1132 |
| 15 | "Artificial neural network*".tw. | 1915 |
| 16 | "Deep Neural Networks".tw. | 494 |
| 17 | "Naïve Bayes".tw. | 3 |
| 18 | "Naive Bayes".tw. | 439 |
| 19 | "Fuzzy Logic".tw. | 677 |
| 20 | "K-Means".tw. | 1371 |
| 21 | "Random Forest".tw. | 956 |
| 22 | "Long Short-Term Memory Networks".tw. | 24 |
| 23 | autoencoder.tw. | 161 |
| 24 | "boltzmann machine".tw. | 85 |
| 25 | "deep belief network".tw. | 56 |
| 26 | "Gradient Boost*".tw. | 184 |
| 27 | AdaBoost.tw. | 174 |
| 28 | "Multilayer Perceptron".tw. | 231 |
| 29 | "Ensemble learning".tw. | 237 |
| 29 | "boltzmann machine".tw. | 340 |
| 30 | "deep belief network".tw. | 371 |
| 31 | "Gradient Boost*".tw. | 6266 |
| 32 | AdaBoost.tw. | 1590 |
| 33 | "Multilayer Perceptron".tw. | 2792 |
| 34 | exp Wearable Electronic Devices/ | 0 |
| 35 | wearable*.tw. | 15723 |
| 36 | "smart watch*".tw. | 102 |
| 37 | smartwatch*.tw. | 842 |
| 38 | acceleromet*.tw. | 21344 |
| 39 | gyroscop*.tw. | 1785 |
| 40 | "inertial sensor".tw. | 455 |
| 41 | "inertial measurement unit*".tw. | 1002 |
| 42 | "fitness band*".tw. | 5 |
| 43 | headband*.tw. | 89 |
| 44 | "head band*".tw. | 35 |
| 45 | wristband*.tw. | 62 |
| 46 | "wrist band*".tw. | 43 |
| 47 | "smart insole*".tw. | 7 |
| 48 | bracelet*.tw. | 78 |
| 49 | Emotiv.tw. | 125 |
| 50 | NeuroSky.tw. | 8 |
| 51 | Mindo.tw. | 7 |
| 52 | Muse.tw. | 656 |
| 53 | Fitbit.tw. | 1023 |
| 54 | Garmin.tw. | 197 |
| 55 | "Polar loop".tw. | 3 |
| 56 | Jawbone.tw. | 683 |
| 57 | Geneactiv.tw. | 188 |
| 58 | Empatica.tw. | 78 |
| 59 | Actigraph.tw. | 3756 |
| 60 | "Apple Watch".tw. | 342 |
| 61 | Amazfit.tw. | 0 |
| 62 | Actiwatch.tw. | 285 |
| 63 | "Mi Band".tw. | 3 |
| 64 | "Oura Ring".tw. | 7 |
| 65 | Vivosmart.tw. | 14 |
| 66 | "Microsoft Band".tw. | 2 |
| 67 | "Actiwave cardio".tw. | 0 |
| 68 | "MindWave Mobile".tw. | 0 |
| 69 | "Galaxy watch".tw. | 6 |
| 70 | Biobeam.tw. | 0 |
| 71 | Stress, Psychological/ | 0 |
| 72 | stress*.tw. | 211897 |
| 73 | distress.tw. | 67486 |
| 74 | Students/ | 105189 |
| 75 | Student*.tw. | 464658 |
| 76 | Pupils.tw. | 15500 |
| 77 | Graduate*.tw. | 71050 |
| 78 | Undergraduate*.tw. | 61384 |
| 79 | Postgraduate*.tw. | 27583 |
| 80 | Collegian*.tw. | 51 |
| 81 | Sophomore*.tw. | 1023 |
| 82 | schoolchildren.tw. | 17941 |
| 83 | 1 or 2 or 3 or 4 or 5 or 6 or 7 or 8 or 9 or 10 or 11 or 12 or 13 or 14 or 15 or 16 or 17 or 18 or 19 or 20 or 21 or 22 or 23 or 24 or 25 or 26 or 27 or 28 or 29 or 30 or 31 or 32 or 33 | 37244 |
| 84 | 34 or 35 or 36 or 37 or 38 or 39 or 40 or 41 or 42 or 43 or 44 or 45 or 46 or 47 or 48 or 49 or 50 or 51 or 52 or 53 or 54 or 55 or 56 or 57 or 58 or 59 or 60 or 61 or 62 or 63 or 64 or 65 or 66 or 67 or 68 or 69 or 70 | 7688 |
| 85 | 71 or 72 or 73 | 258344 |
| 86 | 74 or 75 or 76 or 77 or 78 or 79 or 80 or 81 or 82 | 677516 |
| 87 | 83 and 84 and 85 and 86 | 25 |
| 88 | limit 87 to (english language and yr="2015 -Current") | 23 |

| **Database** | **Query** | **Results** |
| --- | --- | --- |
| **Scopus** | ( TITLE-ABS-KEY ( "Artificial Intelligence" OR "Machine Learning" OR "Deep Learning" OR "supervised learning" OR "unsupervised learning" OR "reinforcement learning" OR "Decision tree" OR "K-Nearest Neighbor*" OR "Support vector machine*" OR "Recurrent neural network*" OR "convolutional neural network*" OR "Artificial neural network*" OR "Deep Neural Networks" OR "Naïve Bayes" OR "Naive Bayes" OR "Fuzzy Logic" OR "K-Means" OR "Random Forest" OR "Long Short-Term Memory Networks" OR autoencoder OR "boltzmann machine" OR "deep belief network" OR "Gradient Boost*" OR "AdaBoost" OR "Multilayer Perceptron" OR "Ensemble learning" ) AND TITLE-ABS-KEY ( wearable* OR "smart watch*" OR smartwatch* OR acceleromet* OR gyroscop* OR "inertial sensor" OR "inertial measurement unit*" OR "fitness band*" OR headband* OR "head band*" OR "wrist band*" OR wristband* OR "smart insole*" OR bracelet* OR emotiv OR neurosky OR mindo OR starlab OR muse OR fitbit OR garmin OR "Polar loop" OR jawbone OR geneactiv OR empatica OR actigraph OR "Apple Watch" OR amazfit OR actiwatch OR "Mi Band" OR "Oura Ring" OR vivosmart OR "Microsoft Band" OR "Actiwave cardio" OR "MindWave Mobile" OR "Galaxy watch" OR biobeam ) AND TITLE-ABS-KEY ( stress* OR distress ) AND TITLE-ABS-KEY ( student* OR pupil* OR graduate* OR undergraduate* OR postgraduate* OR collegian* OR sophomore* OR schoolchildren ) ) AND PUBYEAR > 2014 AND PUBYEAR < 2024 | 49 |
| **IEEE Xplore** | ("Abstract":"Artificial Intelligence" OR "Abstract":"Machine Learning" OR "Abstract":"Deep Learning" OR "Abstract":"supervised learning" OR "Abstract":"unsupervised learning" OR "Abstract":"reinforcement learning" OR "Abstract":"Decision tree" OR "Abstract":"K-Nearest Neighbor*" OR "Abstract":"Support vector machine*" OR "Abstract":"Recurrent neural network*" OR "Abstract":"convolutional neural network*" OR "Abstract":"Artificial neural network*" OR "Abstract":"Deep Neural Networks" OR "Abstract":"Naïve Bayes" OR "Abstract":"Naive Bayes" OR "Abstract":"Fuzzy Logic" OR "Abstract":"K-Means" OR "Abstract":"Random Forest" OR "Abstract":"Long Short-Term Memory Networks" OR "Abstract":autoencoder OR "Abstract":"boltzmann machine" OR "Abstract":"deep belief network" OR "Abstract":"Gradient Boost*" OR "Abstract":"AdaBoost" OR "Abstract":"Multilayer Perceptron" OR "Abstract":"Ensemble learning") AND ("Abstract":wearable OR "Abstract":"smart watch" OR "Abstract":smartwatch OR "Abstract":acceleromet OR "Abstract":gyroscop OR "Abstract":"inertial sensor" OR "Abstract":"inertial measurement unit" OR "Abstract":"fitness band" OR "Abstract":headband OR "Abstract":"head band" OR "Abstract":"wrist band" OR "Abstract":wristband OR "Abstract":"smart insole" OR "Abstract":bracelet OR "Abstract":wearables OR "Abstract":"smart watches" OR "Abstract":smartwatches OR "Abstract":acceleromets OR "Abstract":gyroscopes OR "Abstract":"inertial sensors" OR "Abstract":"inertial measurement units" OR "Abstract":"fitness bands" OR "Abstract":headbands OR "Abstract":"head bands" OR "Abstract":"wrist bands" OR "Abstract":wristbands OR "Abstract":"smart insoles" OR "Abstract":bracelets OR "Abstract":Emotiv OR "Abstract":NeuroSky OR "Abstract":Mindo OR "Abstract":StarLab OR "Abstract":Muse OR "Abstract":Fitbit OR "Abstract":Garmin OR "Abstract":"Polar loop" OR "Abstract":Jawbone OR "Abstract":Geneactiv OR "Abstract":Empatica OR "Abstract":Actigraph OR "Abstract":"Apple Watch" OR "Abstract":Amazfit OR "Abstract":Actiwatch OR "Abstract":"Mi Band" OR "Abstract":"Oura Ring" OR "Abstract":Vivosmart OR "Abstract":"Microsoft Band" OR "Abstract":"Actiwave cardio" OR "Abstract":"MindWave Mobile" OR "Abstract":"Galaxy watch" OR "Abstract":Biobeam) AND ("Abstract":stress OR "Abstract":distress) AND ("Abstract":Student OR "Abstract":Students OR "Abstract":Pupil OR "Abstract":Pupils OR "Abstract":Graduate OR "Abstract":Graduates OR "Abstract":Undergraduate OR "Abstract":Undergraduates OR "Abstract":Postgraduate OR "Abstract":Postgraduates OR "Abstract":Collegian OR "Abstract":Collegians OR "Abstract":Sophomore OR "Abstract":Sophomores OR "Abstract":schoolchildren) | 23 |
| **ACM Digital library** | [[Abstract: "artificial intelligence"] OR [Abstract: "machine learning"] OR [Abstract: "deep learning"] OR [Abstract: "supervised learning"] OR [Abstract: "unsupervised learning"] OR [Abstract: "reinforcement learning"] OR [Abstract: "decision tree"] OR [Abstract: "k-nearest neighbor*"] OR [Abstract: "support vector machine*"] OR [Abstract: "recurrent neural network*"] OR [Abstract: "convolutional neural network*"] OR [Abstract: "artificial neural network*"] OR [Abstract: "deep neural networks"] OR [Abstract: "naïve bayes"] OR [Abstract: "naive bayes"] OR [Abstract: "fuzzy logic"] OR [Abstract: "k-means"] OR [Abstract: "random forest"] OR [Abstract: "long short-term memory networks"] OR [Abstract: autoencoder] OR [Abstract: "boltzmann machine"] OR [Abstract: "deep belief network"] OR [Abstract: "gradient boost*"] OR [Abstract: "adaboost"] OR [Abstract: "multilayer perceptron"] OR [Abstract: "ensemble learning"]] AND [[Abstract: wearable*] OR [Abstract: "smart watch*"] OR [Abstract: smartwatch*] OR [Abstract: acceleromet*] OR [Abstract: gyroscop*] OR [Abstract: "inertial sensor"] OR [Abstract: "inertial measurement unit*"] OR [Abstract: "fitness band*"] OR [Abstract: headband*] OR [Abstract: "head band*"] OR [Abstract: "wrist band*"] OR [Abstract: wristband*] OR [Abstract: "smart insole*"] OR [Abstract: bracelet*] OR [Abstract: emotiv] OR [Abstract: neurosky] OR [Abstract: mindo] OR [Abstract: starlab] OR [Abstract: muse] OR [Abstract: fitbit] OR [Abstract: garmin] OR [Abstract: "polar loop"] OR [Abstract: jawbone] OR [Abstract: geneactiv] OR [Abstract: empatica] OR [Abstract: actigraph] OR [Abstract: "apple watch"] OR [Abstract: amazfit] OR [Abstract: actiwatch] OR [Abstract: "mi band"] OR [Abstract: "oura ring"] OR [Abstract: vivosmart] OR [Abstract: "microsoft band"] OR [Abstract: "actiwave cardio"] OR [Abstract: "mindwave mobile"] OR [Abstract: "galaxy watch"] OR [Abstract: biobeam]] AND [[Abstract: stress*] OR [Abstract: distress]] AND [[Abstract: student*] OR [Abstract: pupil*] OR [Abstract: graduate*] OR [Abstract: undergraduate*] OR [Abstract: postgraduate*] OR [Abstract: collegian*] OR [Abstract: sophomore*] OR [Abstract: schoolchildren]] | 19 |
| **Google Scholar** | ("Artificial Intelligence" OR "Machine Learning" OR "Deep Learning" OR "Decision tree" OR "Support vector machine*") AND (Wearable* OR smartwatch* OR wristband* OR Emotiv OR Muse OR Fitbit OR Actiwatch) AND (Stress* OR distress) AND (Student*) | 100 |
