# Supplementary material for: The Performance of Wearable AI in Detecting Stress Among Students: Systematic Review and Meta-Analysis
Source: J Med Internet Res. 2024 Jan 31;26:e52622. doi: 10.2196/52622 (PMC10867751; doi:10.2196/52622)
Supplement: Multimedia Appendix 3 [file jmir_v26i1e52622_app3.docx]

| **Extracted data** | **Definition** |
| --- | --- |
| **Study Characteristics** |  |
| Author | The first author of the study. |
| Year of publication | The year in which the study was published. |
| Type of publication | The venue where the study was published: peer-reviewed journal articles, book chapters, dissertations, or conference proceedings |
| Country of publication | The country where the study was published. |
| **Participant characteristics** |  |
| Number of participants | What is the number of participants from which the data was collected? |
| Mean age | What is the mean age of the participants? |
| Age range | What is the range of age of the participants? |
| Female percentage | What is the female percentage of the participants? |
| Education level | What is the education level of the participants (School, College, Bachelor, Master, PhD)? |
| **Wearable devices characteristics** |  |
| Status of the wearable device | Is the wearable device a prototype (non-commercial) or is it an already available commercial device (e.g., Fitbit, Apple Watch)? |
| Name of the wearable device | What is the name of the wearable device (e.g., Fitbit, Empatica, ApplyWatch, ActiWatch, etc..)? |
| Type of the wearable device | What is the type of wearable device (e.g., smart band, smart watch, smart glasses, smart clothes, smart socks, smart shoes, etc.)? |
| Placement of the wearable device | Where the wearable device is worn during the experiment in paper or normally (wrist, chest, head, ears, forehead, eyes, fingers, foot, etc..)? |
| Duration of wearing WD | How long was the wearable device worn? |
| **AI** **Characteristics** |  |
| Problem-solving approaches | What is the problem-solving approach that the algorithm follows (Classification, regression, clustering)? |
| Number of classes | For classification problems, how many classes do the AI models detect? |
| AI algorithm used | What are the main AI algorithms/models (e.g., RF, SVM, ANN, CNN, RNN, DNN, k-NN, MLP, DBN, DBM, DPN BN, CRT, DT, LASSO, LR, MFA, MLR, MDL, NB, NN, NSC, RBFN) used in the paper? |
| Aim of AI algorithm | What was the algorithm used for (Detection or Prediction)? Detecting the current state of stress or predicting stress in the future. |
| Dataset size | What is the dataset size used for developing (training & testing) the algorithm? |
| Data sources | What is the type of data (e.g., WD-based data, self-reported data, non-WD-based data) that was used for developing the algorithm? |
| Data types | What is the data that was used for developing the algorithm? |
| Number of features | How many features were used to develop the model? |
| Stress induction method | What is the stimulator that was used to raise the stress level (exam, Stroop, homework, lecture, presentation)? |
| Ground truth | How the actual status (e.g., diagnosis) of the user was confirmed (questionnaire (PHQ-9), interview, test, etc..)? |
| Type of validation | What is the approach that was used to validate the developed algorithm (e.g., Training-test split, K-fold cross-validation, Nested Cross-Validation, Leave One Out cross-validation, Apparent validation, external validation)? |
| Performance measures used | What are the measures used to assess the performance of the algorithm (accuracy, sensitivity (recall), specificity, precision, AUC, MAE, RMSE, R2, etc...)? |
| **Results of studies** |  |
| Number of cases | What is the number of students with stress whose data was used to test the model? |
| Number of controls | What is the number of students without stress whose data was used to test the model? |
| Confusion Matrix | What are the confusion matrix estimates for the highest estimate for each algorithm: true positive, true negative, false positive, false negative |
| Highest accuracy | What is the highest accuracy in the results? |
| Highest sensitivity | What is the highest sensitivity in the results? |
| Highest specificity | What is the highest specificity in the results? |
| Highest F1 score | What is the highest F1 score in the results? |
| Highest RMSE | What is the highest RMSE in the results? |
| Highest MAE | What is the highest MAE in the results? |
