# Supplementary material for: The Performance of Wearable AI in Detecting Stress Among Students: Systematic Review and Meta-Analysis
Source: J Med Internet Res. 2024 Jan 31;26:e52622. doi: 10.2196/52622 (PMC10867751; doi:10.2196/52622)
Supplement: Multimedia Appendix 4 [file jmir_v26i1e52622_app4.docx]

Multimedia Appendix 4: The Modified Version of QUADAS-2

| **1. Participants** | **Signaling questions** | **Explanation** |
| --- | --- | --- |
|  | 1.1 Was a consecutive or random sample of patients enrolled? | -Yes: if a consecutive or random sample of eligible patients was enrolled.  - No: if patients were selected by convenience;  - Unclear: if the study did not report the manner in which participants were enrolled. |
|  | 1.2 Did the study avoid inappropriate exclusions? | - Yes: If inclusion and exclusion of participants were appropriate, so participants correspond to unselected participants of interest.  - No: If participants are included who would already have been identified as having the outcome and so are no longer participants at suspicion of disease (diagnostic studies),  or if specific subgroups are excluded that may have altered the performance of the prediction model for the intended target population.  - Unclear: When there is no information on whether inappropriate inclusions or exclusions took place. |
|  | 1.3 Was the sample size sufficient? | - Yes: For model validation studies, if the number of participants is ≥100, OR having a number of samples ≥100 in cases where more than one sample is recorded for each participant, with the input to the ML algorithm at the sample level, not the subject level.  - No: For model validation studies, if the number of participants with the outcome is <100, AND the number of samples is <100, in cases where more than one sample is recorded for each participant, and the input to the ML algorithm is at the sample level  - Unclear: For model development studies, no information on the number of candidate predictor parameters or number of participants  with the outcome, such that the EPV cannot be calculated.  For model validation studies, no information on the number of participants with the outcome. |
|  | 1.4 Was there a balance in the number of patients between the subgroups (stressed vs. nonstressed)? | - Yes: if the percentage of participants in any group is 66.7 or less of the sample (≤2/3).  - No: if the percentage of participants in any group is more than 66.7 of the sample (>2/3).  - Unclear: If no information was provided regarding the number of participants in the groups. |
|  | **Risk-of-bias assessment:** Could the selection of participants have introduced bias? | - Low risk of bias: If the answer to all signaling questions is ‘Yes’ then the risk of bias can be considered low. If one or more of the answers is ‘No’, the judgment could still be low risk of bias, but specific reasons why the risk of bias can be considered low should be provided.  - High risk of bias: If the answer to any of the signaling questions is “No” there is a potential for bias, except if defined at low risk of bias above.  - Unclear risk of bias: If relevant information is missing for all or some of the signaling questions, and none of the answers to signaling questions is judged to put this domain at high risk of bias. |
|  | **Concerns regarding applicability:** Are there concerns that the included participants and setting do not match the review question? | - Low concern for applicability: If the spectrum of participants (in- and exclusion criteria, setting, prior testing) matches the pre-stated requirements in the review question  - High concern for applicability: If the spectrum of participants does not fully match the pre-stated requirements in the review question  - Unclear concern for applicability: If there is insufficient information available to make a judgment about the applicability |
| **2. Index test (AI algorithms)** | 2.1 Were the AI models described in detail? | -Yes: if the model details were provided such as outputs, epoch, all intermediate layers and connections, pooling, normalization, regularization, and activation in the layers, etc.  or if a previously published model is  employed, the paper must cite a reference that meets the preceding standards and fully describe every modification made to the model.  -No: if only the model’s name was reported in the paper, or the study reported some information but other important information still missing. |
|  | 2.2 Were all features (predictors) used in the model clearly identified? | -Yes: If all features (e.g., heart rate, inter-beat interval, heart rate variation, number of sleep hours, etc.) used in each model were reported.  -No: If any features used in any model were not reported.  Or all features used in the paper were identified but it was not clear which features were used in each model. |
|  | 2.3 Were features assessed in the same way for all participants? | Please notice some studies used different wearable devices to collect the data.  -Yes: If the assessment of features assessment were similar for all participants.  -No: If different definitions were used for the same predictor or if predictors requiring subjective interpretation were assessed by differently experienced assessors.  Unclear: If there is no information on how predictors were defined or assessed. |
|  | 2.4 Were features collected without knowledge of outcome data (stress status)? | - Yes: If outcome information was stated as not used during feature assessment or was clearly not (yet) available to those assessing features.  - No: If it is clear that outcome information was used when assessing predictors.  - Unclear: No information on whether features were assessed without knowledge of outcome information. |
|  | **Risk-of-bias assessment:** Could the conduct or interpretation of the index test have introduced bias? | - Low risk of bias: If the answer to all signaling questions is ‘Yes’ then the risk of bias can be considered low. If one or more of the answers is ‘No’, the judgment could still be low risk of bias, but specific reasons why the risk of bias can be considered low should be provided e.g., the use of objective predictors not requiring subjective interpretation.  - High risk of bias: If the answer to any of the signaling questions is “No” there is a potential for bias, except if defined at low risk of bias above.  - Unclear risk of bias: If relevant information is missing for all or some of the signaling questions, and none of the answers to signaling questions is judged to put this domain at high risk of bias. |
|  | **Concerns regarding applicability:** Are there concerns that the definition, assessment, or timing of the index test in the model does not match the review question? | - Low concern for applicability: Definition, assessment, and timing of predictors match the review question.  - High concern for applicability: Definition, assessment, or timing of predictors were different from the review question.  - Unclear concern for applicability: If relevant information about the predictors is not reported. |
| **3. Reference Standard (Ground truth)** | 3.1 Was the reference standard likely to correctly classify the outcome (e.g., stressed vs. non-stressed)? | Is the used tool appropriate?  Were the assessors/annotators qualified?  Yes: If the study:   - used well-recommended tools (e.g. PSS questionnaire). - Context: if experiment is well described in terms of the design of the stressful events, timing of the events, etc. - Used unsupervised learning to generate labels.   -No: if the outcome was assessed using only one question (e.g., how stressed do you feel today?).  OR Unknown questionnaire with reliability and validity of <0.70 or unknown reliability and validity.  OR an interview was conducted by unqualified assessors such as students.  - Unclear: If no information was provided about the reference standard |
|  | 3.2 Was the outcome defined and determined in a similar way for all participants? | - Yes: If outcomes were defined and determined in a similar way for all participants.  - No: If outcomes were clearly defined and determined in a different way for some participants.  - Unclear: No information on whether outcomes were defined or determined in a similar way for all participants. |
|  | 3.3 Was the outcome determined without knowledge of predictor information? | - Yes: If predictor information was not known when determining the outcome status, or outcome status determination is clearly reported as determined without knowledge of predictor information.  - No: If it is clear that predictor information was used when determining the outcome status.  - Unclear: No information on whether the outcome was determined without knowledge of predictor information. |
|  | 3.4 Was there an appropriate interval between the index test and the reference standard? | Check the period in which the reference standard assesses symptoms of stress. For example, PSS assesses symptoms of stress experienced over the past month. When such tool is used, then the interval between the index test and reference standard should not be more than one month.  - Yes: If the time interval between predictor assessment and outcome determination was appropriate to enable the correct type and representative number of relevant outcomes to be recorded, or if no information on the time interval is required to allow a representative number of the relevant outcome occur or if predictor assessment and outcome determination were from information taken within an appropriate time interval.  - No: If the time interval between predictor assessment and outcome determination is too short or too long to enable the correct type and representative number of relevant outcomes to  be recorded.  - Unclear: If no information was provided on the time interval between predictor assessment and outcome determination. |
|  | **Risk-of-bias assessment:** Could the reference standard, its conduct, or its interpretation have introduced bias? | - Low risk of bias: If the answer to all signaling questions is ‘Yes’ then the risk of bias can be considered low. If one or more of the answers is ‘No’, the judgment could still be low risk of bias, but specific reasons why the risk of bias can be considered low should be provided e.g., when the outcome was  determined with knowledge of predictor information but the outcome assessment did not require much interpretation by the assessor (e.g., death regardless of cause).  - High risk of bias: If the answer to any of the signaling questions is “No” there is a potential for bias, except if defined at low risk of bias above.  - Unclear risk of bias: If relevant information is missing for all or some of the signaling questions, and none of the answers to signaling questions is judged to put this domain at high risk of bias. |
|  | **Concerns regarding applicability:** Are there concerns that the outcome definition, timing, or determination do not  match the review question? | - Low concern for applicability: Outcome definition, timing, and method of determination defines the outcome as intended by the review question.  -High concern for applicability: Choice of outcome definition, timing, and method of outcome determination defines another outcome as intended by the review question.  - Unclear concern for applicability: If relevant information about the outcome, timing, and method of determination is not reported. |
| **4.** **Analysis** | 4.1 Were all participants included in the analysis? | - Yes: If all participants enrolled in the study are  included in the data analysis.  - No: If some or a subgroup of participants are  inappropriately excluded from the analysis.  - Unclear: No information on whether all enrolled participants are included in the analysis. |
|  | 4.2 Was data preprocessing carried out appropriately? | - Yes: If there are no missing values of predictors or outcomes and the study explicitly reports that participants are not excluded on the basis of missing data, or if missing values are handled using multiple imputation.  - No: If participants with missing data are omitted from the analysis, or if the method of handling missing data is clearly flawed, e.g., missing indicator method or inappropriate use of last value carried forward, or if the study had no explicit mention of methods to handle missing data.  - Unclear: If there is insufficient information to determine if the method of handling missing data is appropriate. |
|  | 4.3 Was the breakdown of the training, validation, and test sets appropriate? | - Yes: if the study used an appropriate validation approach.  - No: if the study used an inappropriate validation approach.  - Unclear: If no information was provided about the validation methods. |
|  | 4.4 Was the performance of the model evaluated appropriately? | - Yes: If the confusion matrix was presented,  Or more than one measure was used and the selected measures were appropriate.  - No: If the confusion matrix was not presented, and only one measure was reported,  Or the selected measures were not appropriate.  - Unclear: If no information was provided on the performance measures |
|  | **Risk-of-bias assessment:** Could the analysis, its conduct, or its interpretation have introduced bias? | - Low risk of bias: If the answer to all signaling questions is ‘Yes’ then the risk of bias can be considered low. If one or more of the answers is ‘No’, the judgment could still be low risk of bias, but specific reasons why the risk of bias can be considered low should be provided.  - High risk of bias: If the answer to any of the signaling questions is “No” there is a potential for bias, except if defined at low risk of bias above.  - Unclear risk of bias: If relevant information is missing for all or some of the signaling questions, and none of the answers to signaling questions is judged to put the analysis at high risk of bias. |
