# Supplementary material for: The Performance of Wearable AI in Detecting Stress Among Students: Systematic Review and Meta-Analysis
Source: J Med Internet Res. 2024 Jan 31;26:e52622. doi: 10.2196/52622 (PMC10867751; doi:10.2196/52622)
Supplement: Multimedia Appendix 6 [file jmir_v26i1e52622_app6.docx]

**Multimedia Appendix 6: Features of Wearable Devices**

| Study [Ref] | Status of WD | Name of WD | Type of WD | Placement of WD | Duration of wearing WD |
| --- | --- | --- | --- | --- | --- |
| Altaf [1] | Commercial | Raqib | Smartwatch | Wrist | NR |
| Aqajari [2] | Commercial | Galaxy Gear S | Smartwatch | Wrist | 14 days |
| Can [3] | Commercial | Galaxy Gear S, S2, S3 & Empatica E4 | Smartwatch, smartband | Wrist | 9 days |
| Coutts [4] | Commercial | Biobeam band | Smartband | Wrist | 84 days |
| de Arriba Pérez [5] | Commercial | Microsoft Smartband 2 | Smartband | Wrist | NR |
| Egilmez [6] | Commercial, non-commercial | LG Urban 2, Polar H7, WGSR | Smartwatch, smartband | Wrist, chest | NR |
| Hasanbasic [7] | Commercial | Bitalino | Electrodes | Chest, palm | NR |
| Islam [8] | Commercial | Fitbit Charge 2 | Smartwatch | Wrist | 7 days |
| Jafarlou [9] | Commercial | Galaxy Watch Active2, Oura ring 2 | Smartwatch, Smart ring | Wrist, finger | 365 days |
| Jiang [10] | NR | NR | Smartband | Wrist | 84 days |
| Moridani [11] | NR | NR | Smartband | Wrist | 38 mins |
| Nagar [12] | Commercial | MindWave | Headset | Head | NR |
| Padmaja [13] | Commercial | Fitbit | Smartband | Wrist | 60 days |
| Sandulescu [14] | Commercial | BioNomadix | Smartband | Wrist, finger | 17 mins |
| Sano [15] | Commercial | Q-sensor, Motion Logger | Smartband | Wrist | 30 days |
| Schmidt [16] | Commercial | Empatica E4 | Smartband | Wrist | 2 hours |
| Silva [17] | Commercial | Microsoft Smartband 2 | Smartband | Wrist | NR |
| Tiwari [18] | Commercial | Mysignals sensor | Electrodes | Chest, shoulder, foot, finger, palm | 35 mins |
| Wu [19] | Commercial | Empatica E4 | Smartband | Wrist | 10-20 mins |

1. Altaf H, Ibrahim SN, Olanrewaju RF, editors. Non invasive stress detection method based on discrete wavelet transform and machine learning algorithms. 2021 IEEE 11th IEEE symposium on computer applications & industrial electronics (ISCAIE); 2021: IEEE.

2. Aqajari SAH, Labbaf S, Hoang Tran P, Nguyen B, Asgari Mehrabadi M, Levorato M, et al. Context-Aware Stress Monitoring using Wearable and Mobile Technologies in Everyday Settings. medRxiv. 2023:2023.04. 20.23288181.

3. Can YS, Chalabianloo N, Ekiz D, Ersoy C. Continuous stress detection using wearable sensors in real life: Algorithmic programming contest case study. Sensors. 2019;19(8):1849.

4. Coutts LV, Plans D, Brown AW, Collomosse J. Deep learning with wearable based heart rate variability for prediction of mental and general health. Journal of Biomedical Informatics. 2020;112:103610.

5. de Arriba Perez F, Santos-Gago JM, Caeiro-Rodríguez M, Iglesias MJF. Evaluation of commercial-off-the-shelf wrist wearables to estimate stress on students. JoVE (Journal of Visualized Experiments). 2018 (136):e57590.

6. Egilmez B, Poyraz E, Zhou W, Memik G, Dinda P, Alshurafa N, editors. UStress: Understanding college student subjective stress using wrist-based passive sensing. 2017 IEEE International Conference on Pervasive Computing and Communications Workshops (PerCom Workshops); 2017: IEEE.

7. Hasanbasic A, Spahic M, Bosnjic D, Mesic V, Jahic O, editors. Recognition of stress levels among students with wearable sensors. 2019 18th International Symposium INFOTEH-JAHORINA (INFOTEH); 2019: IEEE.

8. Islam TZ, Liang PW, Sweeney F, Pragner C, Thiagarajan JJ, Sharmin M, et al., editors. College life is hard!-shedding light on stress prediction for autistic college students using data-driven analysis. 2021 IEEE 45th Annual Computers, Software, and Applications Conference (COMPSAC); 2021: IEEE.

9. Jafarlou S, Lai J, Azimi I, Mousavi Z, Labbaf S, Jain RC, et al. Objective prediction of next-day’s affect using multimodal physiological and behavioral data: Algorithm development and validation study. JMIR Formative Research. 2023;7(1):e39425.

10. Jiang J-Y, Chao Z, Bertozzi AL, Wang W, Young SD, Needell D, editors. Learning to predict human stress level with incomplete sensor data from wearable devices. Proceedings of the 28th ACM International conference on information and knowledge management; 2019.

11. Moridani M, Mahabadi Z, Javadi N. Heart rate variability features for different stress classification. Bratislavské lékarské listy. 2020;121(9):619-27.

12. Nagar P, Sethia D, editors. Brain Mapping Based Stress Identification Using Portable EEG Based Device. 2019 11th International Conference on Communication Systems & Networks (COMSNETS); 2019 7-11 Jan. 2019.

13. Padmaja B, Prasad VVR, Sunitha KVN, Reddy NCS, Anil CH, editors. DetectStress: A Novel Stress Detection System Based on Smartphone and Wireless Physical Activity Tracker. First International Conference on Artificial Intelligence and Cognitive Computing; 2019 2019//; Singapore: Springer Singapore.

14. Sandulescu V, Andrews S, Ellis D, Bellotto N, Mozos OM, editors. Stress Detection Using Wearable Physiological Sensors. Artificial Computation in Biology and Medicine; 2015 2015//; Cham: Springer International Publishing.

15. Sano A, Taylor S, McHill AW, Phillips AJ, Barger LK, Klerman E, et al. Identifying Objective Physiological Markers and Modifiable Behaviors for Self-Reported Stress and Mental Health Status Using Wearable Sensors and Mobile Phones: Observational Study. J Med Internet Res. 2018 Jun 8;20(6):e210. PMID: 29884610. doi: 10.2196/jmir.9410.

16. Schmidt P, Reiss A, Duerichen R, Marberger C, Laerhoven KV. Introducing WESAD, a Multimodal Dataset for Wearable Stress and Affect Detection. Proceedings of the 20th ACM International Conference on Multimodal Interaction; Boulder, CO, USA: Association for Computing Machinery; 2018. p. 400–8.

17. Silva E, Aguiar J, Reis LP, Sá JOE, Gonçalves J, Carvalho V. Stress among Portuguese Medical Students: the EuStress Solution. Journal of medical systems. 2020 Jan 2;44(2):45. PMID: 31897774. doi: 10.1007/s10916-019-1520-1.

18. Tiwari S, Agarwal S. A Shrewd Artificial Neural Network-Based Hybrid Model for Pervasive Stress Detection of Students Using Galvanic Skin Response and Electrocardiogram Signals. Big data. 2021 Dec;9(6):427-42. PMID: 34851743. doi: 10.1089/big.2020.0256.

19. Wu Y, Daoudi M, Amad A, Sparrow L, d'Hondt F, editors. Unsupervised learning method for exploring students' mental stress in medical simulation training. Companion Publication of the 2020 International Conference on Multimodal Interaction; 2020.
