# Supplementary material for: The Performance of Wearable AI in Detecting Stress Among Students: Systematic Review and Meta-Analysis
Source: J Med Internet Res. 2024 Jan 31;26:e52622. doi: 10.2196/52622 (PMC10867751; doi:10.2196/52622)
Supplement: Multimedia Appendix 7 [file jmir_v26i1e52622_app7.docx]

**Multimedia Appendix 7: Features of AI Algorithms**

| Study ^Ref^ | Problem solving approach | Number of classes | AI algorithm | Dataset size | Dataset source | Data type | Number of features | Stress inducer | Ground truth assessment | Validation approach |
| --- | --- | --- | --- | --- | --- | --- | --- | --- | --- | --- |
| Altaf [1] | Classification | 2 | BayesNet, KNN, LogR, MLP, NB, SVM | 30 | WD-based | Heart rate data | 8 | Arithmetic tasks | Unsupervised algorithm | K-fold, training-test split |
| Aqajari [2] | Classification | 2 | KNN, RF, XGBoost | 591 | WD-based, self-reported, non-WD based | Activity data, heart rate data, location, respiratory rate data, smartphone usage data, weather data | 23 | NA | Self-reported questionnaire | K-fold |
| Can [3] | Classification | 3 | KNN, LDA, LogR, MLP, SVM | NR | WD-based, self-reported | Activity data, EDA data, heart rate data, skin temperature | 22 | Contest, lectures | Context, self-reported questionnaire | K-fold |
| Coutts [4] | Classification | 2 | LSTM | NR | WD-based, self-reported | Heart rate data | 12 | Exams | Self-reported questionnaire | Training-test split |
| de Arriba Pérez [5] | Classification, regression | 3 | DT, KNN, NB, RF, SVM, Zero R | NR | WD-based | EDA data, heart rate data, skin temperature | 12 | Arithmetic tasks, exams, lectures, physical stress, Stroop test | Context | K-fold |
| Egilmez [6] | Classification | 2 | LogR, NB, RF, SVM | NR | WD-based, self-reported | EDA data, heart rate data | 110 | Arithmetic tasks, physical stress, singing, Stroop test, video games | Context, self-reported questionnaire | K-fold, LOOCV |
| Hasanbasic [7] | Classification | 3 | DT, Ensemble, KNN, LDA, SVM | 323 | WD-based | EDA data, heart rate data | 12 | Exams, presentations | Context | K-fold |
| Islam [8] | Classification | 2 | ANN, KNN, LogR, SVM | 471 | WD-based, self-reported, non-WD based | Activity data, heart rate data, light exposure, sleep data, sound exposure | 16 | NA | Self-reported questionnaire | K-fold |
| Jafarlou [9] | Classification | 2 | KNN, MLP, RF, SVM | NR | WD-based, self-reported, non-WD based | Activity data, EDA data, heart rate data, location, respiratory rate data, sleep data, smartphone usage data, wearther data | 52 | NA | Self-reported questionnaire | K-fold |
| Jiang [10] | Classification | 2, 5 | AB, ANN, DT, KNN, LinR, RF, SVM, THAN | 855 | WD-based, self-reported | Activity data, sleep data | NR | NA | Self-reported questionnaire | Training-test split |
| Moridani [11] | Classification | 2 | CNN, KNN, MLP, SVM | NR | WD-based | Heart rate data | 8 | Arithmetic tasks, physical stress, Stroop test, watching horror movie | Context | K-fold |
| Nagar [12] | Classification | 2, 3 | KNN, SVM | NR | WD-based | EEG data | 4 | NA | Self-reported questionnaire | K-fold, training-test split |
| Padmaja [13] | Classification | 2 | DT, NB | NR | WD-based, self-reported, non-WD based | Activity data, heart rate data, sleep data, social interaction | 25 | NA | Self-reported questionnaire | NR |
| Sandulescu [14] | Classification | 2 | SVM | NR | WD-based | EDA data, heart rate data | 4 | Arithmetic tasks, public speaking task | Context | Training-test split |
| Sano [15] | Classification | 2 | LinR, SVM | NR | WD-based, self-reported, non-WD based | Activity data, demographics, EDA data, location, personality types, skin temperature, sleep data, smartphone usage data, social interaction | 498 | NA | Self-reported questionnaire | Nested |
| Schmidt [16] | Classification | 2, 3 | AB, DT, KNN, LDA, RF | 1178 | WD-based, non-WD based | Activity data, EDA data, EMG data, heart rate data, respiratory rate data, temperature data, | 92 | Arithmetic tasks, public speaking task | Context | LOOCV |
| Silva [17] | Classification | 2 | ANN, KNN, LogR, NB, RF, SVM | 960 | WD-based, self-reported | Heart rate data | 13 | Exams | Self-reported questionnaire | Training-test split |
| Tiwari [18] | Classification | 4 | ANN, BAG, GB, KNN, LogR, RF, SVM | 84 | WD-based, self-reported | EDA data, heart rate data | 14 | Arithmetic tasks, Stroop test | Self-reported questionnaire | K-fold |
| Wu [19] | Clustering | 2 | K-means | 169 | WD-based | EDA data, heart rate data | 15 | Medical simulation training | Context | NA |
| AB: AdaBoost; ANN: Artificial Neural Network; BAG: Bagging classifiers; BayesNet: Bayes network; CNN: Convolutional Neural Network; DT: Decision tree, EDA: Electrodermal activity; EEG: Electroencephalogram; EMG: Electromyography; IBK: Instant Based Learner; GB: gradient boosting; KNN: K-Nearest Neighbors; LDA: Linear Discriminant Analysis; LinR: Linear regression; LogR: Logistic regression; LOOCV: Leave-One-Out Cross-Validation; LSTM: Long Short-Term Memory; MLP: Multilayer perceptron; NA: Not applicable; NB: Naive Bayes; NR: Not reported; RF: Random Forest; SVM: Support Vector Machine; SVR: Support Vector regressor; THAN: Temporally Hierarchical Attention Network; WD: Wearable device; XGBoost: extreme gradient boosting | | | | | | | | | | |

1. Altaf H, Ibrahim SN, Olanrewaju RF, editors. Non invasive stress detection method based on discrete wavelet transform and machine learning algorithms. 2021 IEEE 11th IEEE symposium on computer applications & industrial electronics (ISCAIE); 2021: IEEE.

2. Aqajari SAH, Labbaf S, Hoang Tran P, Nguyen B, Asgari Mehrabadi M, Levorato M, et al. Context-Aware Stress Monitoring using Wearable and Mobile Technologies in Everyday Settings. medRxiv. 2023:2023.04. 20.23288181.

3. Can YS, Chalabianloo N, Ekiz D, Ersoy C. Continuous stress detection using wearable sensors in real life: Algorithmic programming contest case study. Sensors. 2019;19(8):1849.

4. Coutts LV, Plans D, Brown AW, Collomosse J. Deep learning with wearable based heart rate variability for prediction of mental and general health. Journal of Biomedical Informatics. 2020;112:103610.

5. de Arriba Perez F, Santos-Gago JM, Caeiro-Rodríguez M, Iglesias MJF. Evaluation of commercial-off-the-shelf wrist wearables to estimate stress on students. JoVE (Journal of Visualized Experiments). 2018 (136):e57590.

6. Egilmez B, Poyraz E, Zhou W, Memik G, Dinda P, Alshurafa N, editors. UStress: Understanding college student subjective stress using wrist-based passive sensing. 2017 IEEE International Conference on Pervasive Computing and Communications Workshops (PerCom Workshops); 2017: IEEE.

7. Hasanbasic A, Spahic M, Bosnjic D, Mesic V, Jahic O, editors. Recognition of stress levels among students with wearable sensors. 2019 18th International Symposium INFOTEH-JAHORINA (INFOTEH); 2019: IEEE.

8. Islam TZ, Liang PW, Sweeney F, Pragner C, Thiagarajan JJ, Sharmin M, et al., editors. College life is hard!-shedding light on stress prediction for autistic college students using data-driven analysis. 2021 IEEE 45th Annual Computers, Software, and Applications Conference (COMPSAC); 2021: IEEE.

9. Jafarlou S, Lai J, Azimi I, Mousavi Z, Labbaf S, Jain RC, et al. Objective prediction of next-day’s affect using multimodal physiological and behavioral data: Algorithm development and validation study. JMIR Formative Research. 2023;7(1):e39425.

10. Jiang J-Y, Chao Z, Bertozzi AL, Wang W, Young SD, Needell D, editors. Learning to predict human stress level with incomplete sensor data from wearable devices. Proceedings of the 28th ACM International conference on information and knowledge management; 2019.

11. Moridani M, Mahabadi Z, Javadi N. Heart rate variability features for different stress classification. Bratislavské lékarské listy. 2020;121(9):619-27.

12. Nagar P, Sethia D, editors. Brain Mapping Based Stress Identification Using Portable EEG Based Device. 2019 11th International Conference on Communication Systems & Networks (COMSNETS); 2019 7-11 Jan. 2019.

13. Padmaja B, Prasad VVR, Sunitha KVN, Reddy NCS, Anil CH, editors. DetectStress: A Novel Stress Detection System Based on Smartphone and Wireless Physical Activity Tracker. First International Conference on Artificial Intelligence and Cognitive Computing; 2019 2019//; Singapore: Springer Singapore.

14. Sandulescu V, Andrews S, Ellis D, Bellotto N, Mozos OM, editors. Stress Detection Using Wearable Physiological Sensors. Artificial Computation in Biology and Medicine; 2015 2015//; Cham: Springer International Publishing.

15. Sano A, Taylor S, McHill AW, Phillips AJ, Barger LK, Klerman E, et al. Identifying Objective Physiological Markers and Modifiable Behaviors for Self-Reported Stress and Mental Health Status Using Wearable Sensors and Mobile Phones: Observational Study. J Med Internet Res. 2018 Jun 8;20(6):e210. PMID: 29884610. doi: 10.2196/jmir.9410.

16. Schmidt P, Reiss A, Duerichen R, Marberger C, Laerhoven KV. Introducing WESAD, a Multimodal Dataset for Wearable Stress and Affect Detection. Proceedings of the 20th ACM International Conference on Multimodal Interaction; Boulder, CO, USA: Association for Computing Machinery; 2018. p. 400–8.

17. Silva E, Aguiar J, Reis LP, Sá JOE, Gonçalves J, Carvalho V. Stress among Portuguese Medical Students: the EuStress Solution. Journal of medical systems. 2020 Jan 2;44(2):45. PMID: 31897774. doi: 10.1007/s10916-019-1520-1.

18. Tiwari S, Agarwal S. A Shrewd Artificial Neural Network-Based Hybrid Model for Pervasive Stress Detection of Students Using Galvanic Skin Response and Electrocardiogram Signals. Big data. 2021 Dec;9(6):427-42. PMID: 34851743. doi: 10.1089/big.2020.0256.

19. Wu Y, Daoudi M, Amad A, Sparrow L, d'Hondt F, editors. Unsupervised learning method for exploring students' mental stress in medical simulation training. Companion Publication of the 2020 International Conference on Multimodal Interaction; 2020.
