# Supplementary material for: The Performance of Wearable AI in Detecting Stress Among Students: Systematic Review and Meta-Analysis
Source: J Med Internet Res. 2024 Jan 31;26:e52622. doi: 10.2196/52622 (PMC10867751; doi:10.2196/52622)
Supplement: Multimedia Appendix 8 [file jmir_v26i1e52622_app8.docx]

**Multimedia Appendix 8: Reviewers’ judgments about each domain in “risk of bias” and "applicability concerns" for each included study**
